# *De novo* sequencing, assembly and characterisation of *Aloe vera* transcriptome and analysis of expression profiles of genes related to saponin and anthraquinone metabolism

**DOI:** 10.1186/s12864-018-4819-2

**Published:** 2018-06-01

**Authors:** Pragati Choudhri, Muniya Rani, Rajender S. Sangwan, Ravinder Kumar, Anil Kumar, Vinod Chhokar

**Affiliations:** 1 Department of Bio and Nano Technology, Guru Jambheshwar University of Science and Technology, Hisar, Haryana 125001 India; 2 Centre of Innovative and Applied Bioprocessing (CIAB), (A National Institute under Department of Biotechnology, Govt. of India), Sector-81 (Knowledge City), Manauli P.O., S.A.S. Nagar, Mohali, Punjab 140306 India

**Keywords:** *Aloe vera*, Next generation sequencing, Transcriptome, *De novo* assembly, Secondary metabolism, Differential gene expression

## Abstract

**Background:**

*Aloe vera* is a perennial, succulent, drought-resistant plant that exhibits many pharmacological characteristics such as wound healing ability against skin burns, anti-ulcer, anti-inflammatory, anti-tumor, anti-viral, anti-hypercholesterolemic, anti-hyperglycemic, anti-asthmatic and much more. Despite great medicinal worth, little genomic information is available on *Aloe vera*. This study is an initiative to explore the full-scale functional genomics of *Aloe vera* by generating whole transcriptome sequence database, using Illumina HiSeq technology and its progressive annotation specifically with respect to the metabolic specificity of the plant.

**Results:**

Transcriptome sequencing of root and leaf tissue of *Aloe vera* was performed using Illumina paired-end sequencing technology. *De novo* assembly of high quality paired-end reads, resulted into 1,61,733 and 2,21,792 transcripts with mean length of 709 and 714 nucleotides for root and leaf respectively. The non-redundant transcripts were clustered using CD-HIT-EST, yielding a total of 1,13,063 and 1,41,310 unigenes for root and leaf respectively. A total of 6114 and 6527 CDS for root and leaf tissue were enriched into 24 different biological pathway categories using KEGG pathway database. DGE profile prepared by calculating FPKM values was analyzed for differential expression of specific gene encoding enzymes involved in secondary metabolite biosynthesis. Sixteen putative genes related to saponin, lignin, anthraquinone, and carotenoid biosynthesis were selected for quantitative expression by real-time PCR. DGE as well as qRT PCR expression analysis represented up-regulation of secondary metabolic genes in root as compared to leaf. Furthermore maximum number of genes was found to be up-regulated after the induction of methyl jasmonate, which stipulates the association of secondary metabolite synthesis with the plant’s defense mechanism during stress. Various transcription factors including bHLH, NAC, MYB were identified by searching predicted CDS against PlantTFdb.

**Conclusions:**

This is the first transcriptome database of *Aloe vera* and can be potentially utilized to characterize the genes involved in the biosynthesis of important secondary metabolites, metabolic regulation, signal transduction mechanism, understanding function of a particular gene in the biology and physiology of plant of this species as well as other species of *Aloe* genus.

**Electronic supplementary material:**

The online version of this article (10.1186/s12864-018-4819-2) contains supplementary material, which is available to authorized users.

## Background

The genus *Aloe* belongs to the family *Xanthorrhoeaceae*, subfamily *Asphodeloideae* [1] consisting of many shrubby tropical/subtropical plant species with succulent and elongated leaves. The genus contains more than 360 species, out of which only four have been reported to exhibit medicinal properties: *Aloe vera, Aloe arborescens, Aloe ferox,* and *Aloe perryi.* Among all the species, *Aloe vera Linne* Synonym *Aloe barbadensis Miller* is considered to be medicinally most potent, therefore, it is most popular [2, 3]. *Aloe vera* shows innumerable medicinal attributes such as skin burn healing [4], anti-inflammatory [5], hepatoprotective [6], anti-tumor [7], anti-ulcer [8], antihypercholesterolemic [9], anti-hyperglycemic [10], anti-asthmatic[11] and antioxidant activities [12]. *Aloe vera* extracts have been demonstrated to be advantageous in the treatment of even AIDS [13]. It has similar anti-aging effects as exhibited by vitamin A derivatives [14].

*Aloe vera* contains numerous active ingredients including anthraquinones, polysaccharides, alkylbenzenes, dehydrabietic acid derivatives, salicylic acid, lectin, carotenoids, lignin, saponins etc. that attribute for its high therapeutic value [15]. Many of the medicinal properties of *Aloe vera* are ascribed to secondary metabolites though they are relatively minor in their concentration in the plant (< 1% dry weight) [16]. *Aloe vera* exhibits antibacterial property because of anthraquinones that behave like tetracycline by blocking the ribosomal A site, thus, interrupting bacterial protein synthesis [17]. Saponins are another important group of secondary metabolites synthesized as defensive compounds against pathogenic microbes and herbivores [18–20]. Saponins present in *Aloe vera* also act as an effective anti-microbial agent against various bacteria, viruses, fungi, and yeasts [21]. *Aloe vera* extract act as an antioxidant due to carotenoids present in it [22]. Lignins present in *Aloe vera* penetrate deep into the skin and help in introducing other medicinal ingredients to penetrate into the skin. Therapeutic effects of *Aloe vera* have not been correlated well with individual metabolite and it remains unknown whether the biological activities of the plant are due to a single component or the collaborative efforts of many components [23].

An increasing trend where consumers tend towards a healthy lifestyle, coupled with the increased use of *Aloe vera* extracts as an ingredient in food, pharmaceutical and cosmetics products, increases its market growth across the globe. International Aloe Science Council (IASC) estimated that the global consumption of *Aloe vera* will surpass 60,720.4 tonnes in 2016, accounting for revenues worth US$ 1.6 billion [24]. Presently, a large industrial sector is attempting to exploit the great biological potential of *Aloe vera*. The biological potential is determined by the various complex interactions between the genome, gene products, and metabolites. Various functional genomics approaches are now emerging as powerful tools to accelerate the comprehensive understanding of the molecular basis of biological functions. Genomics approaches have also proved to be significant tools in identifying transcription factors, and candidate genes involved in the plant’s secondary metabolism [25]. Next-generation sequencing technology has revolutionized the genomics field by providing a rapid, cost- effective and efficient gene sequencing data, enabling the identification of genes related to metabolic pathways, especially in non-model plants for which no reference genome is available; for instance; American ginseng, [26] *Eucalyptus,* [27] rubber tree [28] and many more. Transcriptome sequencing using Illumina mRNA-Seq reads has proved to be an efficient approach in many plants for which transcriptome data is validated and analyzed as well, including *Eucalyptus,* [27] blackberry, [29] *Uncaria,* [30] *Lolium*, [31] orphan, [32] sesame, [33] alfalfa, [34] sweet potato, [35] *Centella,* [36] *Ocimum* [37] pear, [38] rose-scented geranium, [39] *Withania somnifera* [40]. The present study used NGS technology for whole transcriptome sequencing of *Aloe vera* root and leaf tissues, performed using Illumina Hi-Seq technology. It provides valuable sequence information from *Aloe vera* root and leaf tissues with special emphasis on genes related to secondary metabolic pathways. Genes related to secondary metabolic pathways were examined for differential expression in root and leaf tissue by quantitative real-time PCR. Real-time PCR comprises a dynamic range, remarkable sensitivity, and sequence-specificity that enables additional independent confirmation of NGS based data [41] Methyl jasmonate treatment was given to the experimental plant to check the relative expression of genes under stress conditions at different time intervals. The transcriptome sequencing and analysis provides a strong platform of genomic sequences, to serve biosynthetic pathway and metabolic engineering programs of basic as well as applied research on *Aloe vera* and catalyze path of its change of use from extracts to molecules.

## Results

### *De novo* transcriptome assembly and validation

**Fig. 1:**
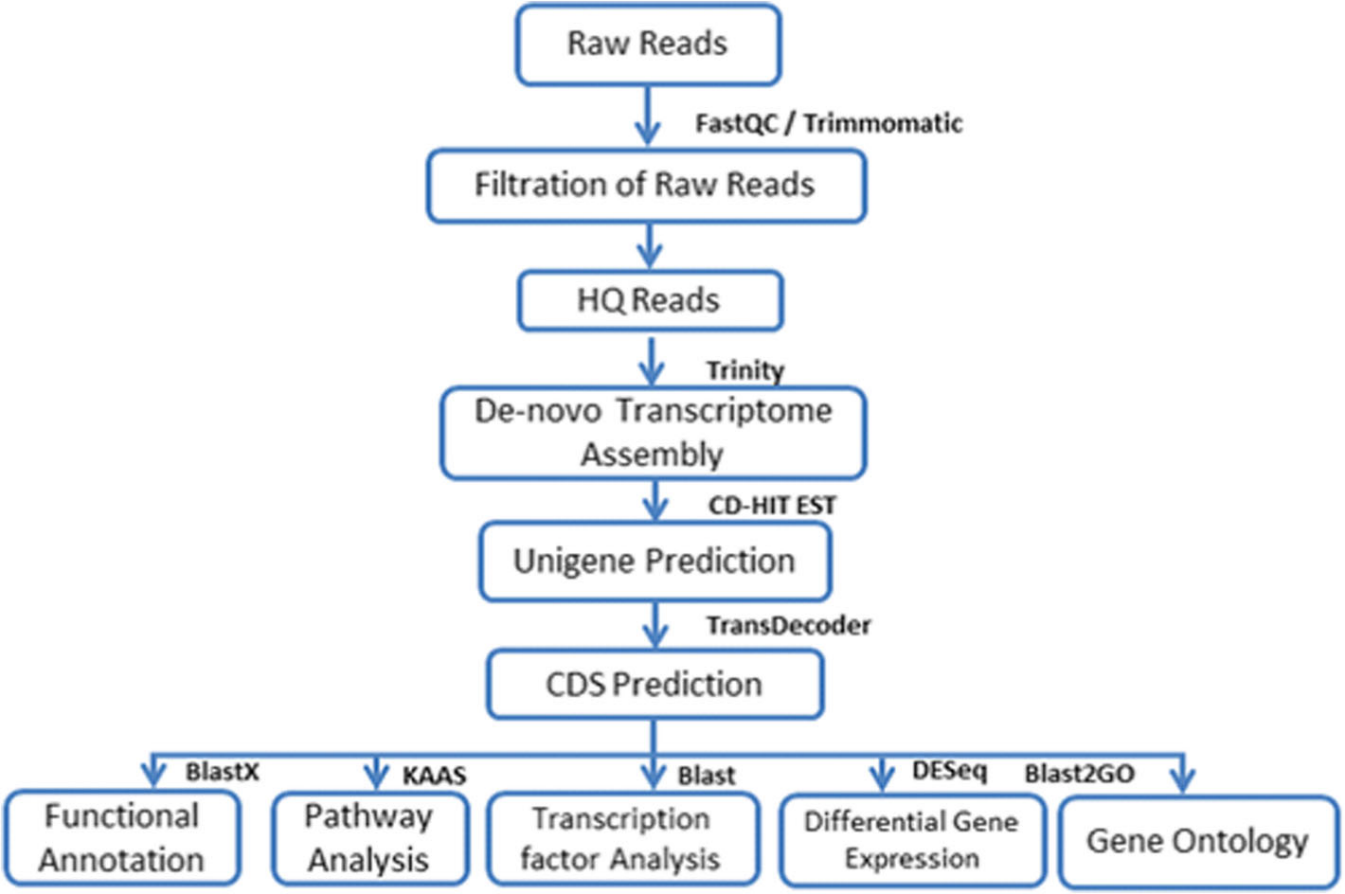
Flow chart: Bioinformatics Analysis Work flow. The figure summarizes the steps undertaken and tools used during *Aloe vera* transcriptome sequencing

Illumina Hi Seq platform sequencing results of cDNAs prepared from total RNA when processed by removing the adapter sequences, ambiguous reads i.e. reads with unknown nucleotides “N” larger than 5%, and low-quality sequences i.e. reads with more than 10% quality threshold, QV < 20 phred score, resulted in high-quality transcriptome data as 51,078,070 (2 × 150 bp; 14,868,243,650 nucleotides), 29,247,010 (2 × 150 bp; 5,907,896,020 nucleotides) paired-end reads (QV > 20) for root and leaf, respectively. The reads obtained were further used for de-novo assembly by Trinity RNA-Seq assembler [42] (Fig. 1: Flow chart: Bioinformatics Analysis Work flow). The analysis resulted into having, 1, 61,733 and 2, 21,792 assembled transcripts for root and leaf with a mean length of 709 and 714 nucleotides for root and leaf respectively (summary of obtained transcript, unigenes and CDS statistics is given in Table 1). The non-redundant transcripts were clustered together using CD-HIT-EST considering 95% identity and query coverage to obtain unigenes. Overall 1,13,063 unigenes for root and 1,41,310 unigenes for leaf have been identified with an average length of 743 bp and 640 bp for root and leaf respectively, including 25,330 unigenes for root and 23,959 unigenes for leaf having sequence size > 1000 bp. Candidate coding regions within unigenes were identified using TransDecoder. A total of 43,443 and 43,178 CDS were obtained for root and leaf with a mean length of 881 bp for root which is somewhat larger than that of leaf (866 bp).

**Table 1:** Summary of Transcript, Unigene and CDS Statistics

Transcripts	Root	Leaf	Unigene	Root	Leaf	CDS	Root	Leaf
Total no.	161,733	221,792	Total no.	113,063	141,310	Total no.	43,443	43,178
Length (bases)	114,692,240	158,472,568	Length (bases)	84,101,877	90,489,454	Length (bases)	38,282,214	37,413,222
Maximum length	15,043	16,503	Maximum length	15,043	16,503	Maximum length	11,679	12,837
Minimum length	201	201	Minimum length	201	201	Minimum length	297	297
Mean length	709	714	Mean length	743	640	Mean length	881	866

### Functional annotation

**Fig. 2:**
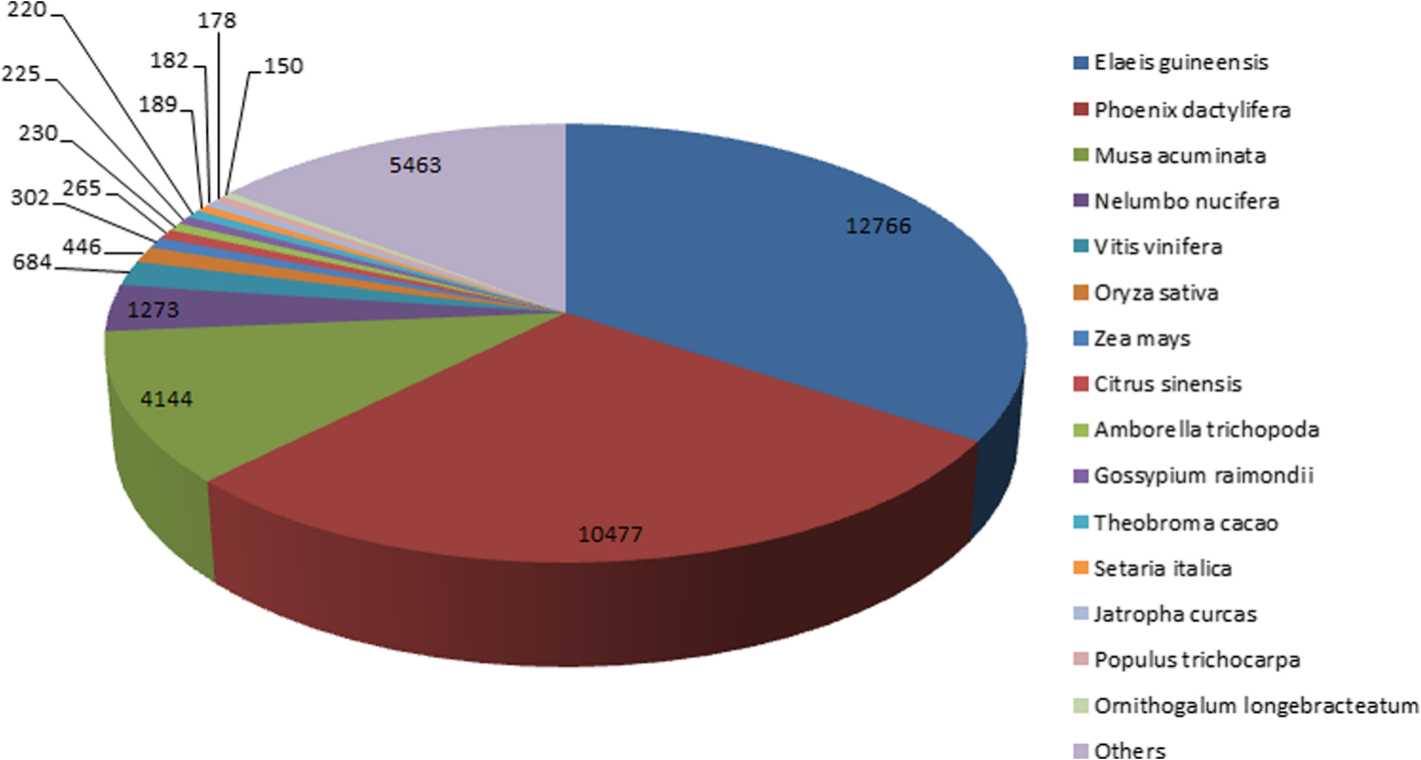
Top blast hit species distribution of root sample. The pie chart represents the number, name and distribution of significant blast hit species with respect to identified CDSs in root sample

**Fig. 3:**
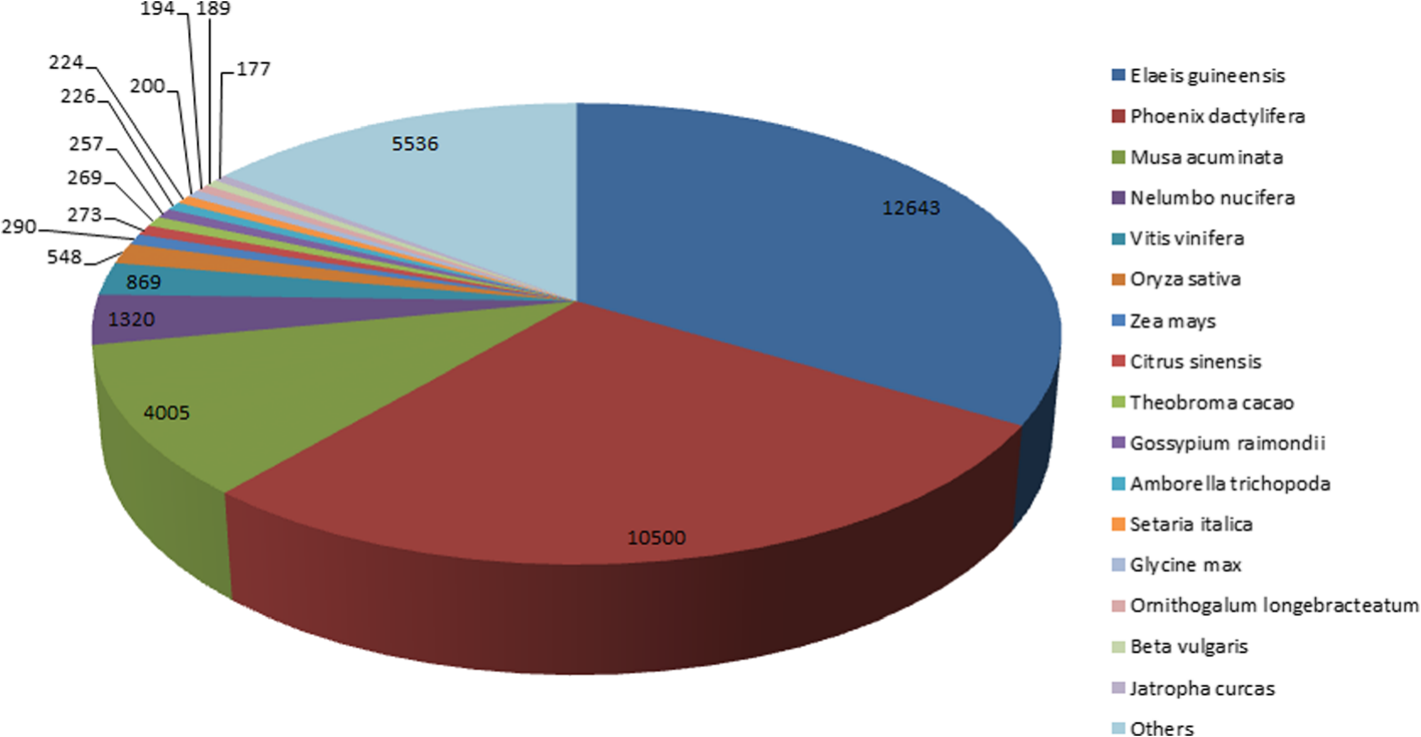
Top blast hit species distribution of leaf sample. The representation of number, name and distribution of significant blast hit species with respect to identified CDSs in leaf sample in the form of pie chart

**Fig. 4:**
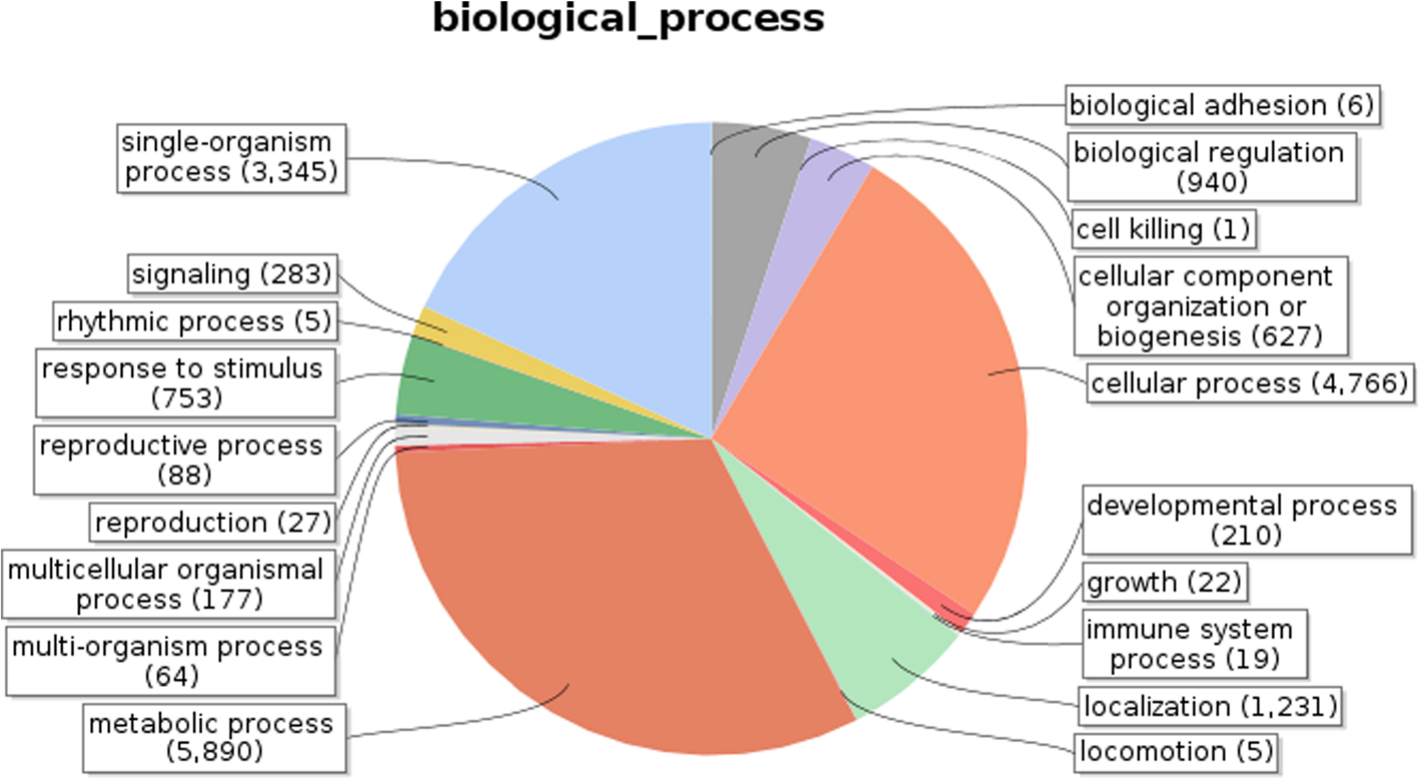
Biological Process GO term distribution of root sample. Figure representing the different biological processes and no. of genes involved in individual biological process and their distribution ratio in form of pie chart for root

**Fig. 5:**
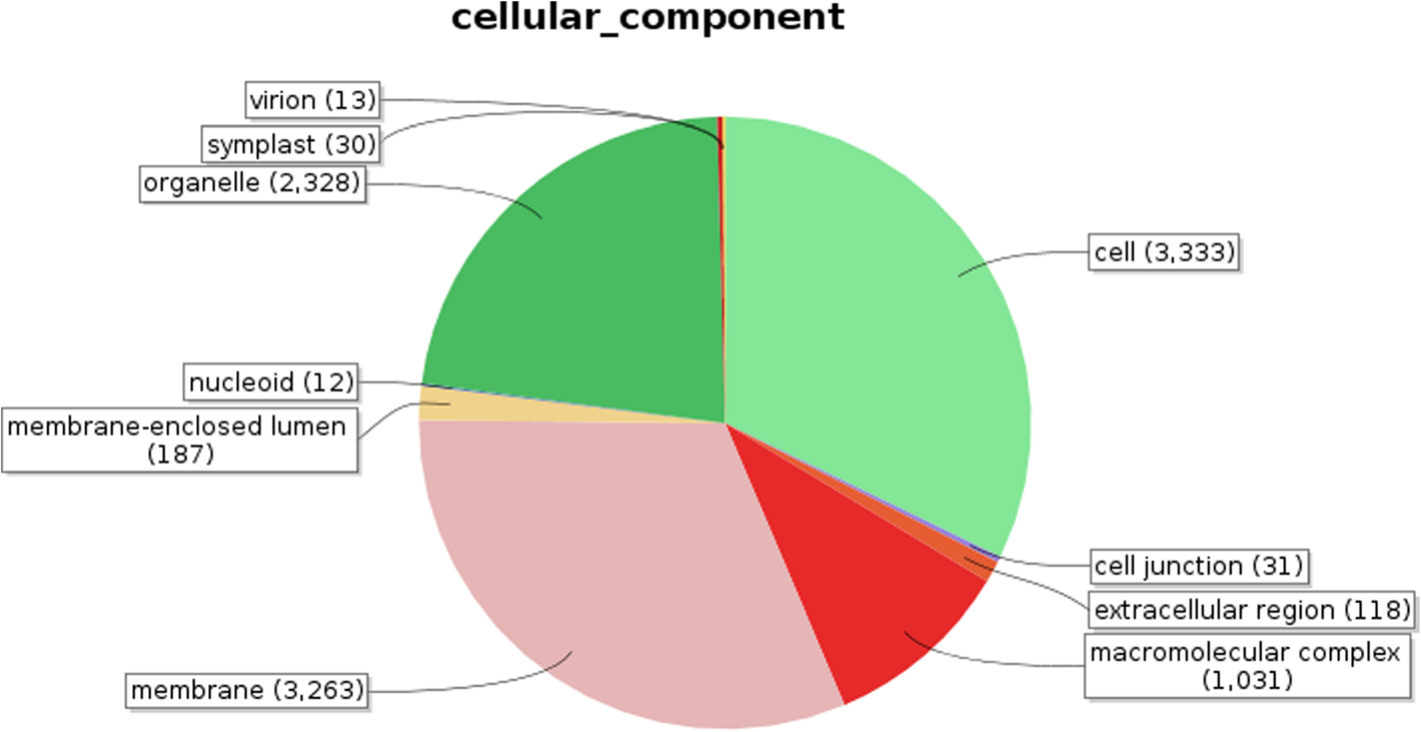
Cellular Component GO term distribution of root sample. The figure represents various cellular processes and distribution of identified genes in different cellular processes in the form of pie chart for root

**Fig. 6:**
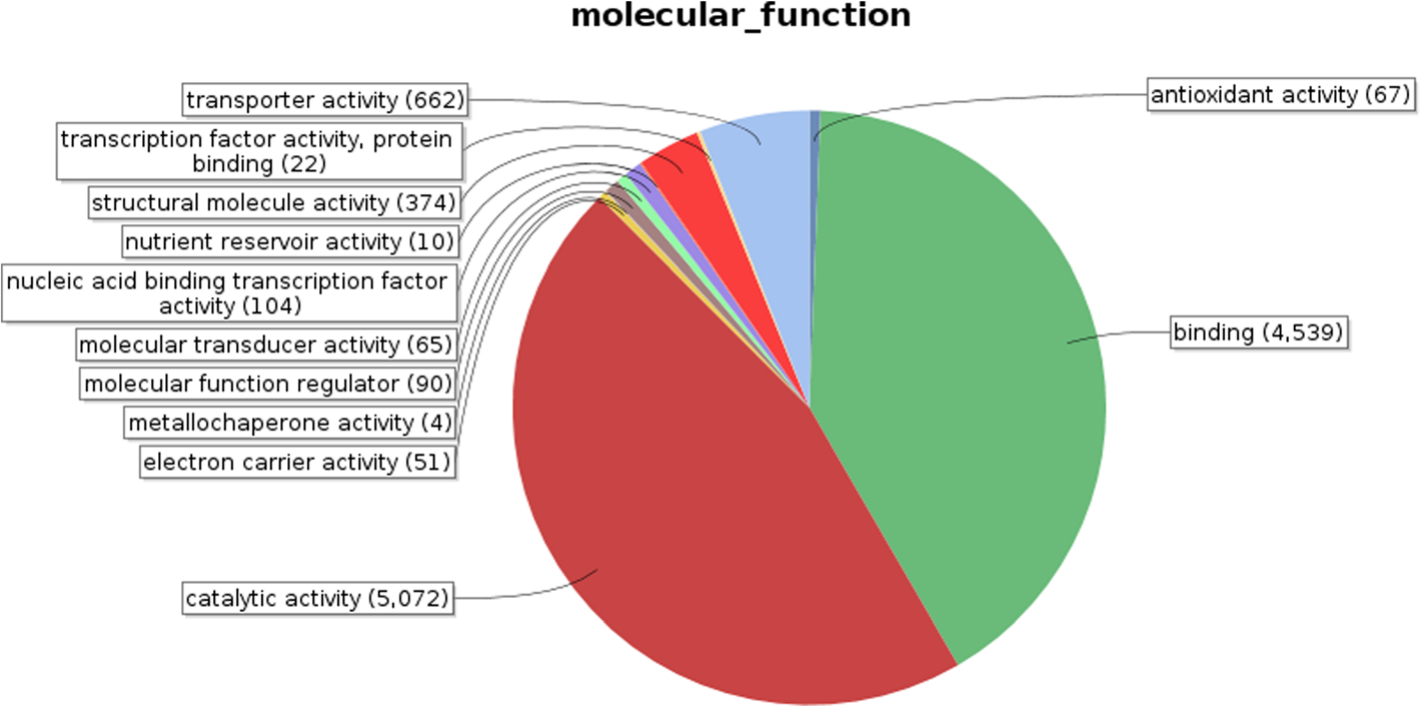
Molecular Function GO term distribution of root sample. Representation of different molecular functions performed by annotated genes and their distribution ratio for root

**Fig. 7:**
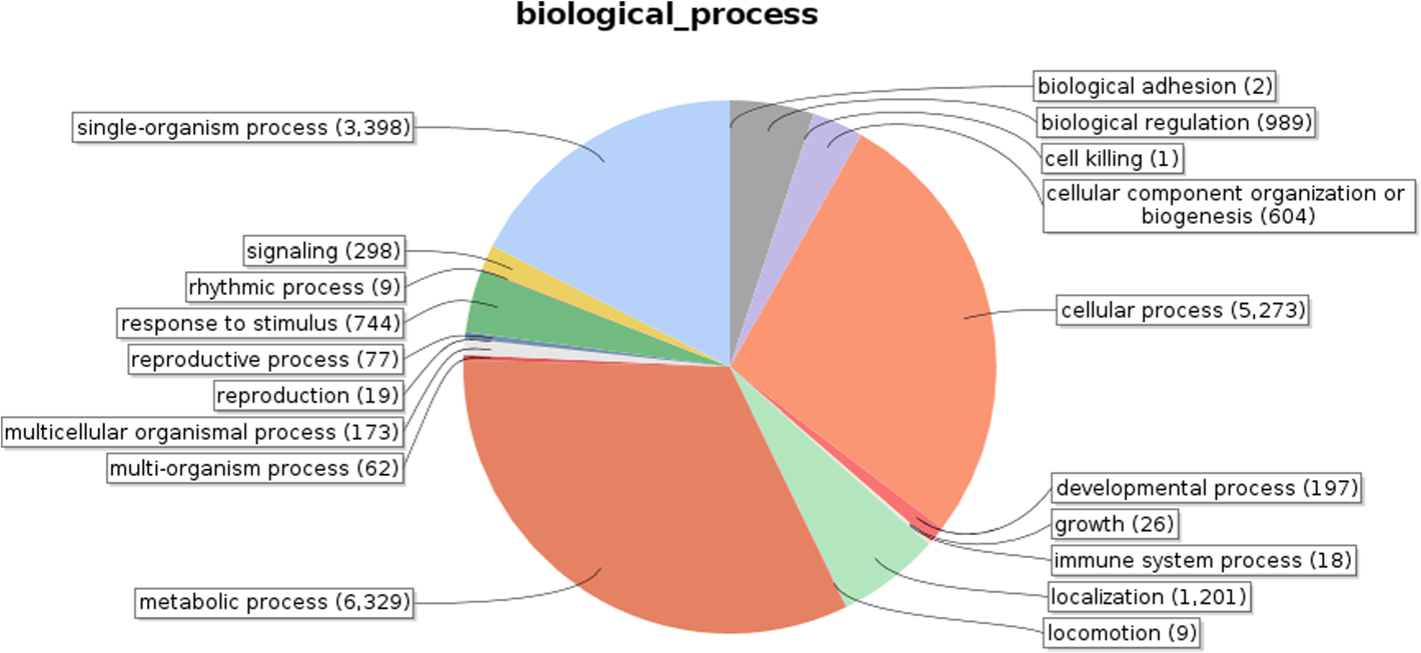
Biological Process GO term distribution of leaf sample. The figure represents different biological process and no. of genes involved in individual process and their distribution ration in pie chart form for leaf

**Fig. 8:**
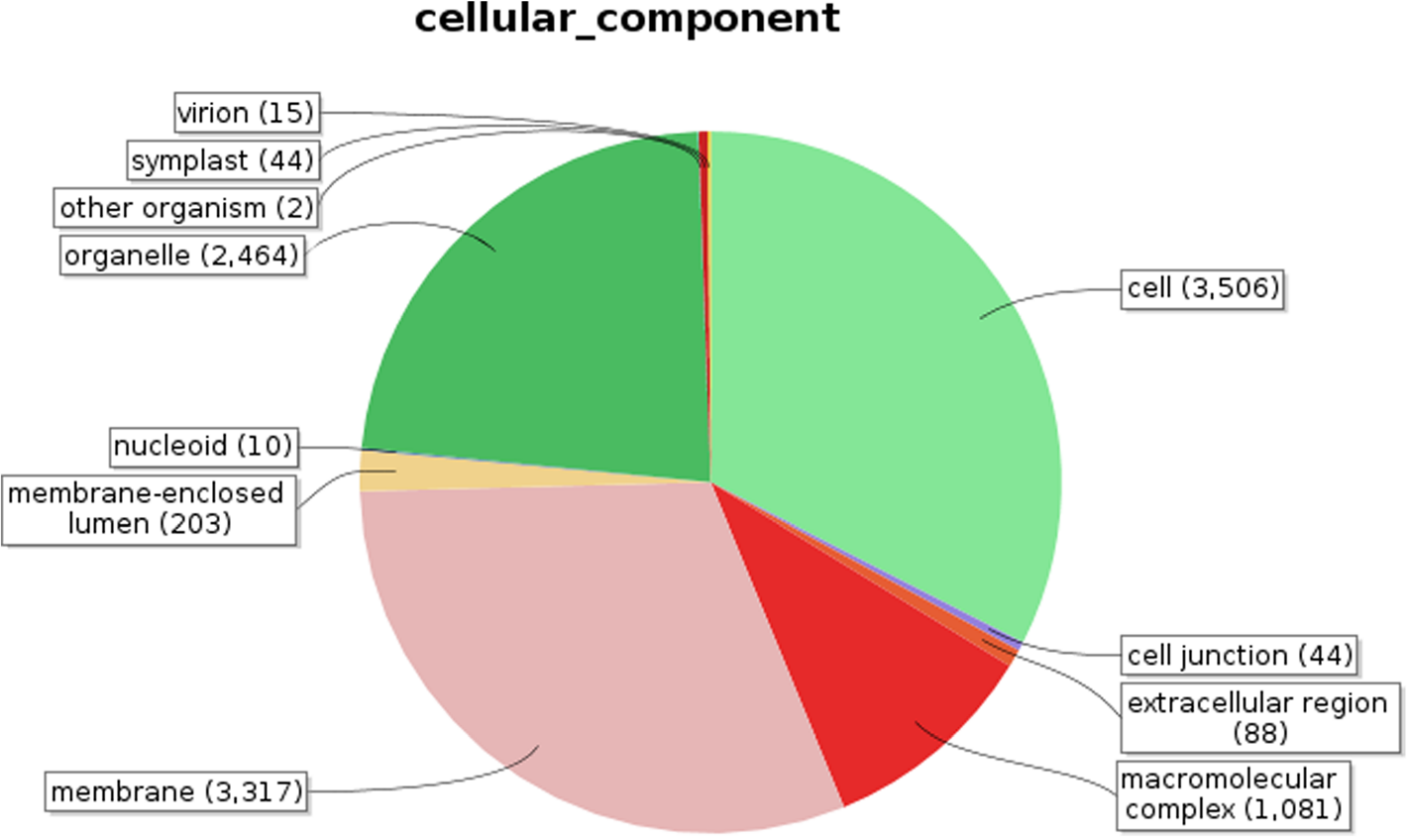
Cellular Component GO term distribution of leaf sample. The figure represents various cellular processes in pie chart form for leaf

**Fig. 9:**
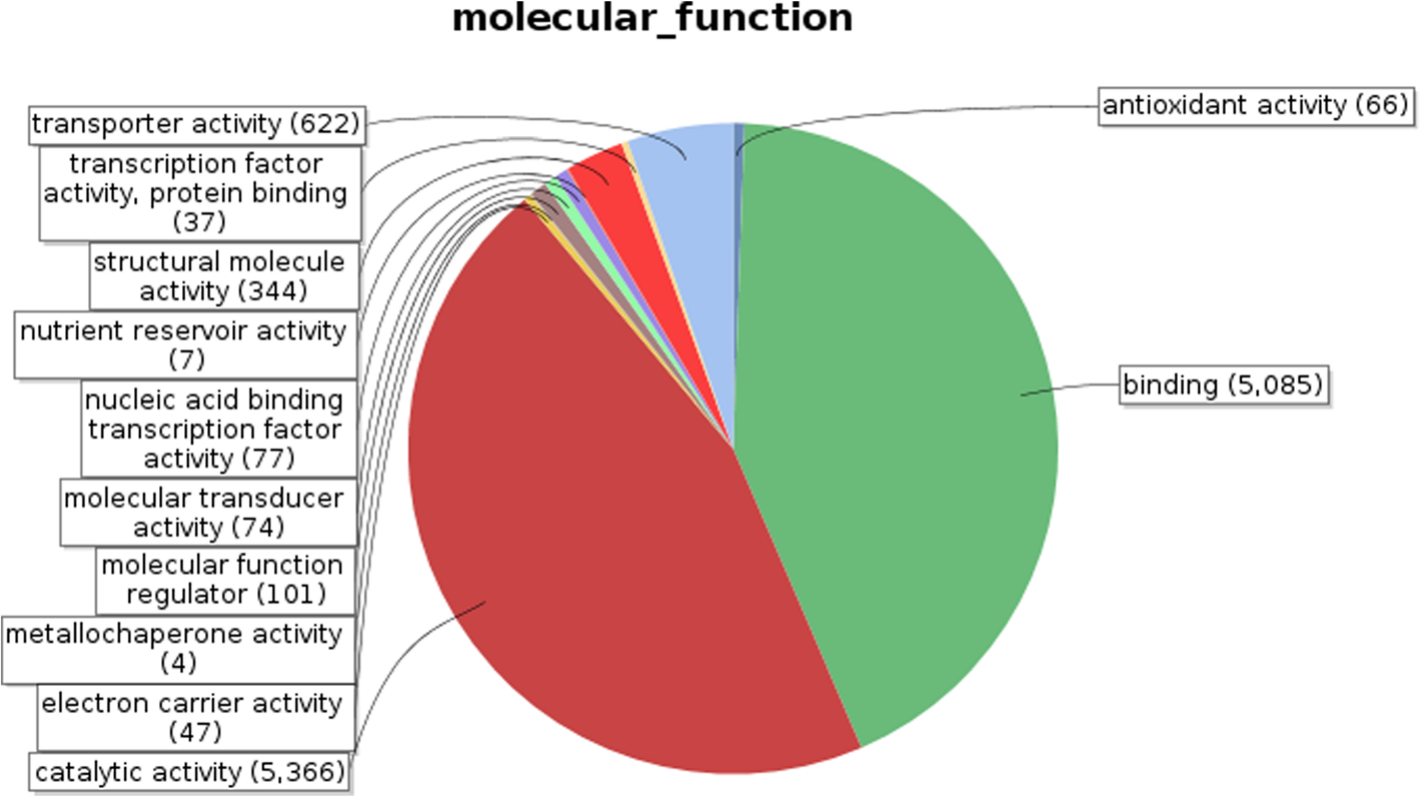
Molecular Function GO term distribution of leaf sample. Representation of different molecular functions performed by annotated genes and their distribution ratio in form of pie chart for leaf sample

**Fig. 10:**
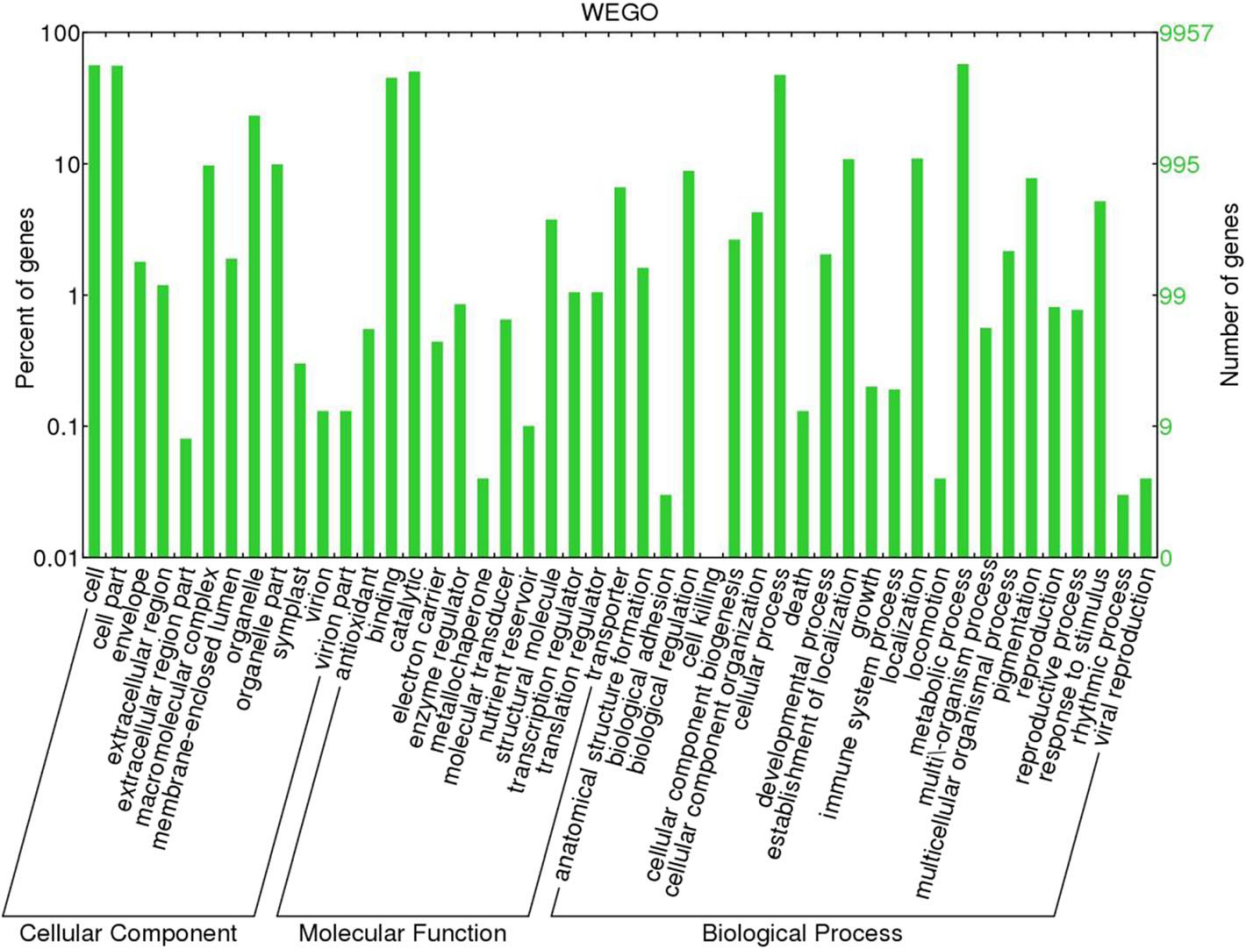
GO Sequence Distribution of root sample. The figure represents the functional distribution of identified CDSs involved in cellular process, molecular function and biological process according to the percentage of genes participating in various functions for root

**Fig. 11:**
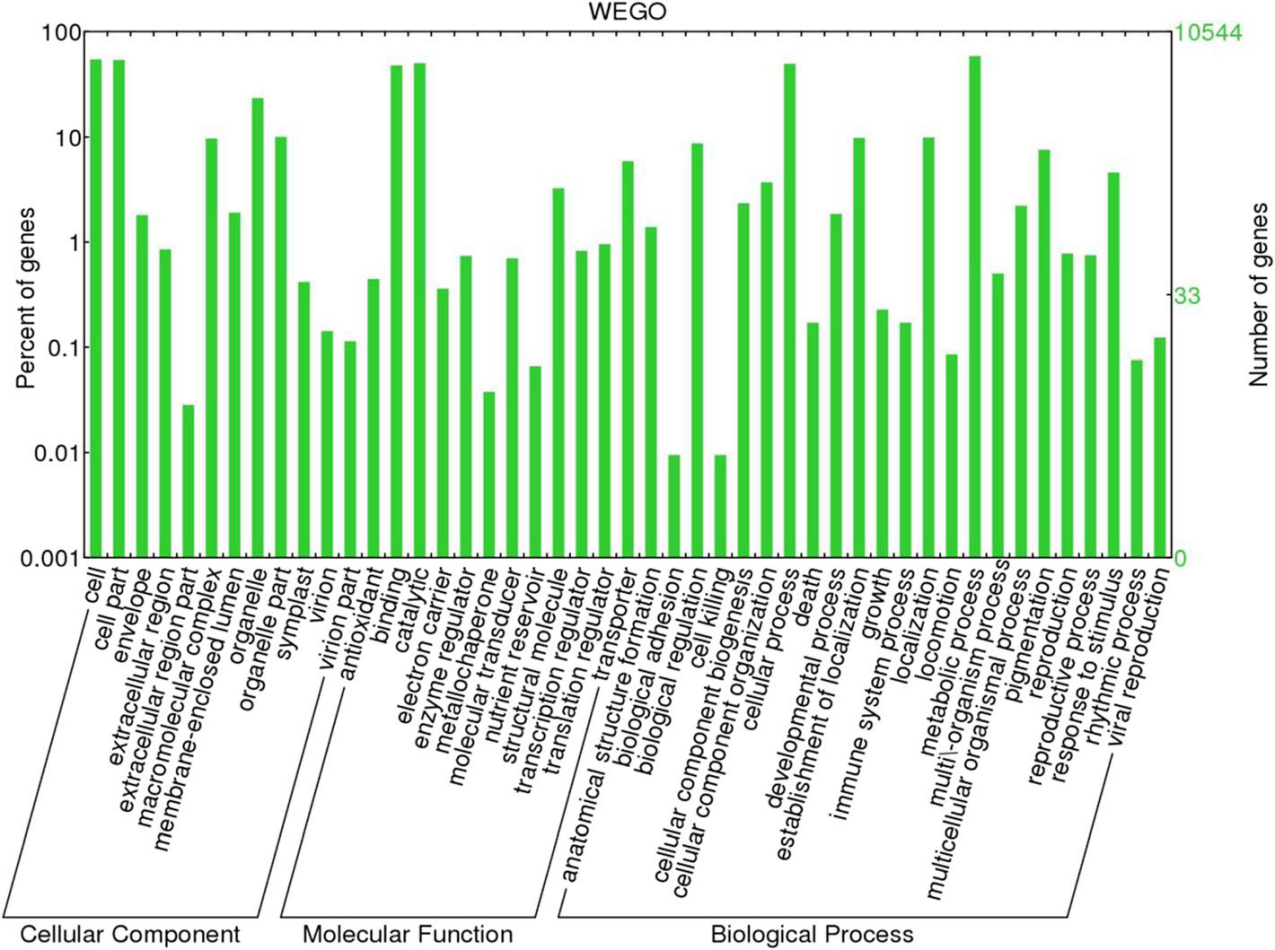
GO Sequence Distribution of leaf sample. The functional distribution of identified CDSs involved in cellular process, molecular function and biological process according to the percentage of genes participating in various functions for leaf

Identified CDS were searched against NCBI non-redundant (Nr) protein database using Basic local alignment search programme BlastX, considering E-value ≤1e-05.BLASTX resulted in the annotation of 37,194 and 37,720 CDS for root and leaf samples respectively. Out of above CDS, 6249 from root and 5458 from leaf had no significant BLAST hit. The majority of hits were found to be against *Elaeis guineensis* (34%) followed by *Phoenix dactylifera* (28%) (Figs. 2 and 3). GO mapping was carried out to assign the functions for BLASTX annotated CDS, using Blast2GO program. BLASTX result accession IDs were used to retrieve UniProt IDs making use of Protein information resources (PIR) like PSD, UniProt, SwissProt, TrEMBL, RefSeq, GenPept and PDB databases. Accession IDs are searched directly in the dbxreftable of GO database. In root sample 18,459 CDS were found to be involved in biological processes (Fig. 4), 10,346 in cellular components (Fig. 5) and 11,060 in molecular functions (Fig. 6) whereas in leaf sample, 19,429 CDS were involved in biological processes (Fig. 7), 10,774 in cellular components (Fig. 8) and 11,830 in molecular functions (Fig. 9). About 35% genes of root and leaf were involved in biological processes, 39% having molecular functions and remaining 26% genes were identified to engage in cellular processes (Figs. 10 and 11). In case of biological processes of root and leaf, a maximum number of genes (5890 genes in root and 6329 genes in leaf) were involved in metabolic processes followed by the genes related to cellular processes (root 4766;leaf 5273) and single-organism process (root 3345;leaf 3398) of the plant. Some other genes were also identified including response to stimulus (root 753;leaf 744), localization (root 1231;leaf 1201), biological regulation (root 940; leaf 989), multicellular organism process (root 177; leaf 173), biogenesis (root 627; leaf 604), signaling (root 283;leaf 298), developmental process (root 210;leaf 197), reproductive process (root 115; leaf 96), growth (root 22; leaf 26), immune system process (root 19; leaf 18), rhythmic process (root 5; leaf 9), locomotion (root 5; leaf 9), biological adhesion (root 6; leaf 2) and cell killing activity (root 1; leaf 1). Cellular component GO term distribution of function tells that mostly genes were involved in cell maintenance (root 3333; leaf 3506), membrane function (root 3263; leaf 3.317) and organelle formation (root 2328; leaf 2464). GO distribution of the genes related to molecular functions showed that maximum genes were referred to catalytic activity (root 5072; leaf 5366; leaf) and binding (root 4539; leaf 5085) followed by genes of the transporter (root 662; leaf 622) and structural molecular activity (root 374; leaf 344). A few other genes related to molecular function, have been annotated including molecular function regulator (root 90; leaf 101), transcription factor to nucleic acid binding (root 104; leaf 77), transcription factor to protein binding (root 22; leaf 37), electron carrier activity (root 51; leaf 47), nutrient reservoir (root 10; leaf 7) and metallochaperone activity (root 4; leaf 4).(Additional file 1: Top 10 most represented GO terms of 3 major GO domains in *Aloe vera* root and leaf).

### Transcription factor analysis

Several transcription factors have been identified by searching the predicted CDS against plant transcription factor database PlantTFdb. Majority of hits were found to be with basic helix loop helix (bHLH) transcription factor, the number being 816 in root tissue and 806 in leaf tissue respectively. Although role of bHLH transcription factor is still unclear in plants. Second highest hits were found to be with NAC (683) in root sample. NAC is the largest family of plant transcription factors related to plant stress responses. A total no. of 706 hits were got against MYB related transcription factors in leaf sample, which are also involved in plant stress responses as well as other biological processes like development, differentiation, defense metabolism etc. Other transcription factors enriched from the transcriptome data were C2H2 (root 554; leaf 487), WRKY(root 520; leaf 596), FAR1 (root 503; leaf 516), B3 (root 455; leaf 486), C3H (root 436; leaf 461), ERF (root 409; leaf 374), bZIP (root 395; leaf 357), G2-like (root 353; leaf 329), ARF (root 228; leaf 319) and S1Fa-like (root 231; leaf 241). TFs, HD-ZIP (leaf 336) and HSF (root 221) were found to be specific for leaf and root respectively (Tables 2 and 3).

**Table 2:** List of Top 15 transcription factors enriched in Root Sample

Transcription factors name	# of Hits
bHLH	816
NAC	683
MYB_related	682
C2H2	554
WRKY	520
FAR1	503
B3	455
C3H	436
ERF	409
MYB	398
bZIP	395
G2-like	353
S1Fa-like	231
ARF	228
HSF	221

**Table 3:** List of Top 15 transcription factors enriched in Leaf Sample

Name Of Transcription Factor	# of Hits
bHLH	806
MYB_related	706
NAC	678
WRKY	596
MYB_related	533
FAR1	516
C2H2	487
B3	486
C3H	461
ERF	374
bZIP	357
HD-ZIP	336
G2-like	329
ARF	319
S1Fa-like	242

### KEGG pathway mapping of CDS

The identified CDS were mapped to reference canonical pathways in KEGG to review the potential involvement of predicted genes in a particular biological pathway. In *Aloe vera* transcriptome KEGG analysis, 6114 CDS for root and 6527 CDS for leaf were found enriched in 24 different KEGG pathway categories. A total of 2902 genes for root and 3139 genes for leaf were functionally assigned for metabolism comprising carbohydrate metabolism (root 527; leaf 560), energy metabolism (root 315; leaf 376), lipid metabolism (root 289; leaf 293), nucleotide metabolism (root 203; leaf 231), amino acid metabolism (root 504; leaf 538), glycan biosynthesis and metabolism (root 126; leaf 130), metabolism of cofactors and vitamins (root 244; leaf 251), metabolism of terpenoids and polyketides (root 131; leaf 134), biosynthesis of other secondary metabolites (root 130; leaf 122) and xenobiotics biodegradation and metabolism (root 66; leaf 69). Genes related to genetic information processing has been assigned for their role in transcription (root 308; leaf 358), translation (root 659; leaf 708), folding, sorting and degradation (root 535; leaf 583), replication and repair (root 186; leaf 180). In root and leaf tissue, 580 and 616 CDS were involved in environmental information processing involving membrane transport **(**root 27; leaf 27) and signal transduction (root 554; leaf 589). One gene was found specific for root working as a signaling molecule and interact to stimulate a signaling cascade. A total of 748 CDS in root and 758 CDS in leaf were found to be involved in cellular processes and 194 genes in root and 192 genes in leaf were found related to environmental adaptation by KEGG analysis (Table 4).

**Table 4:** KEGG Pathway classification of Root and Leaf Sample CDS

Metabolism	Leaf	Root
Overview	435	367
Carbohydrate metabolism	560	527
Energy metabolism	376	315
Lipid metabolism	293	289
Nucleotide metabolism	231	203
Amino acid metabolism	385	355
Metabolism of other amino acids	153	149
Glycan biosynthesis and metabolism	130	126
Metabolism of cofactors and vitamins	251	244
Metabolism of terpenoids and polyketides	134	131
Biosynthesis of other secondary metabolites	122	130
Xenobiotics biodegradation and metabolism	69	66
Genetic Information Processing		
Transcription	358	308
Translation	708	659
Folding, sorting and degradation	583	535
Replication and repair	180	186
Environmental Information Processing		
Membrane transport	27	27
Signal transduction	589	554
Signaling molecules and interaction	0	1
Cellular Processes		
Transport and catabolism	381	382
Cell motility	58	55
Cell growth and death	241	247
Cellular community	71	64
Organismal systems		
Environmental adaptation	192	194

### Potential gene identification related to secondary metabolism from KEGG pathway mapping

In our research, several putative genes related to saponin, lignin, anthraquinone, carotenoid and phenylpropanoid biosynthesis have been identified from *Aloe vera* root and leaf tissue by KEGG Pathway functional annotation. A total of 171 CDS from root encoding 80 enzymes and 165 CDS from leaf encoding 77 enzymes were identified which are involved in different secondary metabolites biosynthesis comprising saponin (root 12; leaf 15), anthraquinone (root 31; leaf 43), lignin (root 25; leaf 23), carotenoid (root 31; leaf 32) and phenylpropanoid pathways (root 72; leaf 52).

Sequencing of *Aloe vera* transcriptome reveals many putative genes (number of different transcripts obtained is given in bracket with each gene) related to saponin biosynthesis pathway including acetyl-CoA acetyltransferase (ACT: root 3; leaf 9), HMG-CoA synthase (HMGS: root 1; leaf 2), HMG-CoA reductase (HMGR: root 2; leaf 4), mevalonate kinase (MVK: root 9; leaf 14), phosphomevalonate kinase (PMVK: root 1; leaf 2), mevalonate-5-diphosphate decarboxylase (MDD: root 1; leaf 1), isopentenyl-PP isomerase (IPP isomerase: root 2; leaf 1), farnesyl diphosphate synthase (FDS: root 1; leaf 1), squalene synthase (SQS: root 1; leaf 1), squalene epoxidase (SQE: root 4; leaf 1), cycloartenol synthase (CAS: root 6; leaf 6), 1-deoxy-D-xylulose-5-phosphate synthase (DOXP: root 9; leaf 10) and 1-deoxy-D-xylulose-5-phosphate reductoisomerase (DOXPR: root 1; leaf 3). From transcriptome sequencing, 61 different transcripts from root and 52 transcripts from leaf that encode UDP glycosyltransferase (UGT) have been obtained. Anthraquinones are another important secondary metabolites present in *Aloe vera.* *De novo* sequencing of *Aloe vera* in this study has provided many unigenes encoding octaketide/polyketide synthase (8 from root and 12 from leaf), aldoketoreductase (25 from root and 22 from leaf) and UDP-glycosyltransferase (61 from root and 52 from leaf) which play a key role in anthraquinone biosynthesis. Enzymes related to lignin biosynthesis viz. L-phenylalanine ammonia-lyase (PAL), caffeoyl CoA O-methyltransferase (CCoAOMT), caffeic acid O-methyltransferase (COMT), 4-coumarate:coenzyme A(CoA) ligase (4CL), cinnamoyl-CoA reductase (CCR), hydroxycinnamoyl transferase (HCT), cinnamate-4-hydroxylase (C4H), 4-coumarate 3-hydroxylase (C3H), ferulate 5-hydroxylase (F5H), cinnamyl alcohol dehydrogenase (CAD) were identified from the *Aloe vera* database by KEGG pathway mapping and the results were then further validated by differential gene expression and real-time expression analysis.

The transcriptomic analysis has provided various genes related to carotenoid biosynthesis including phytoene synthase (root 10; leaf 4), 15-cis-phytoene desaturase (root 2; leaf 2), zeta-carotene isomerase (root 1; leaf 2), prolycopene isomerase (root 4; leaf 1), lycopene beta-cyclase (root 1; leaf 1), lycopene epsilon-cyclase (root 1; leaf 1), zeaxanthin epoxidase (root 18; leaf 15), violaxanthin de-epoxidase (root 7; leaf 9) and abscisic-aldehyde oxidase (root 1; leaf 1) (Table 5).

**Table 5:** CDS present in specific pathways

Leaf Sample			
Name Of Pathway	Annotated CDS	No. of Enzyme	KO IDS
Saponin Pathway	15	10	10
Anthraquinone Pathway	43	24	24
Lignin Pathway	23	10	10
Carotenoid Pathway	32	18	18
Phenylpropanoid Pathway	52	15	15
Root Sample			
Name Of Pathway	Annotated CDS	No. of Enzyme	KO IDS
Saponin Pathway	12	10	10
Anthraquinone Pathway	31	24	24
Lignin Pathway	25	10	10
Carotenoid Pathway	31	19	19
Phenylpropanoid Pathway	72	17	17

### Differential gene expression analysis and real-time expression analysis of transcripts involved in secondary metabolism

**Fig. 12:**
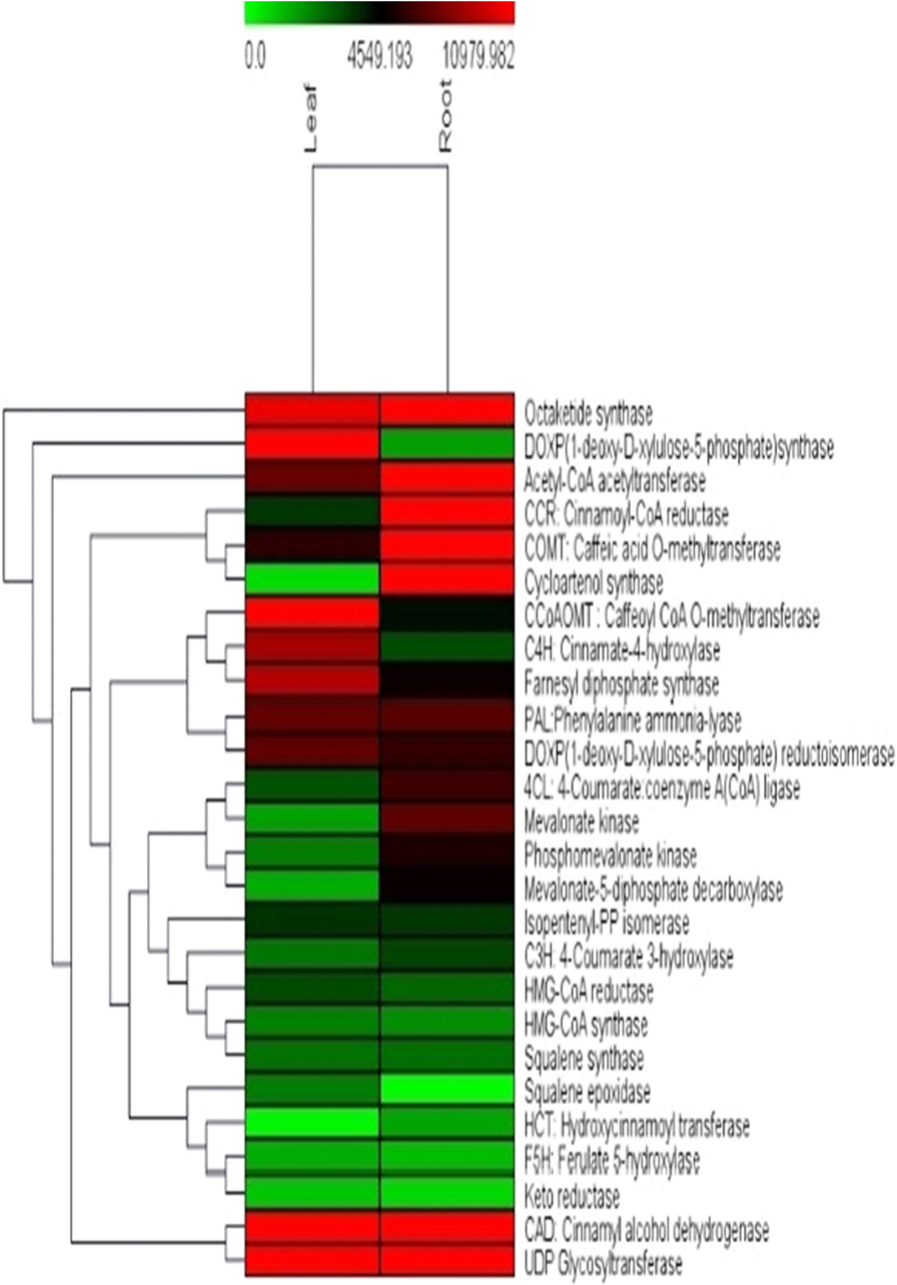
Heat Map of differentially expressed genes of leaf vs. root involved in lignin, saponin and anthraquinone biosynthesis pathway in *Aloe vera.* Green colour in the map represents low expression of the unigenes whereas red colour in the map represents high expression of the unigenes

**Fig. 13:**
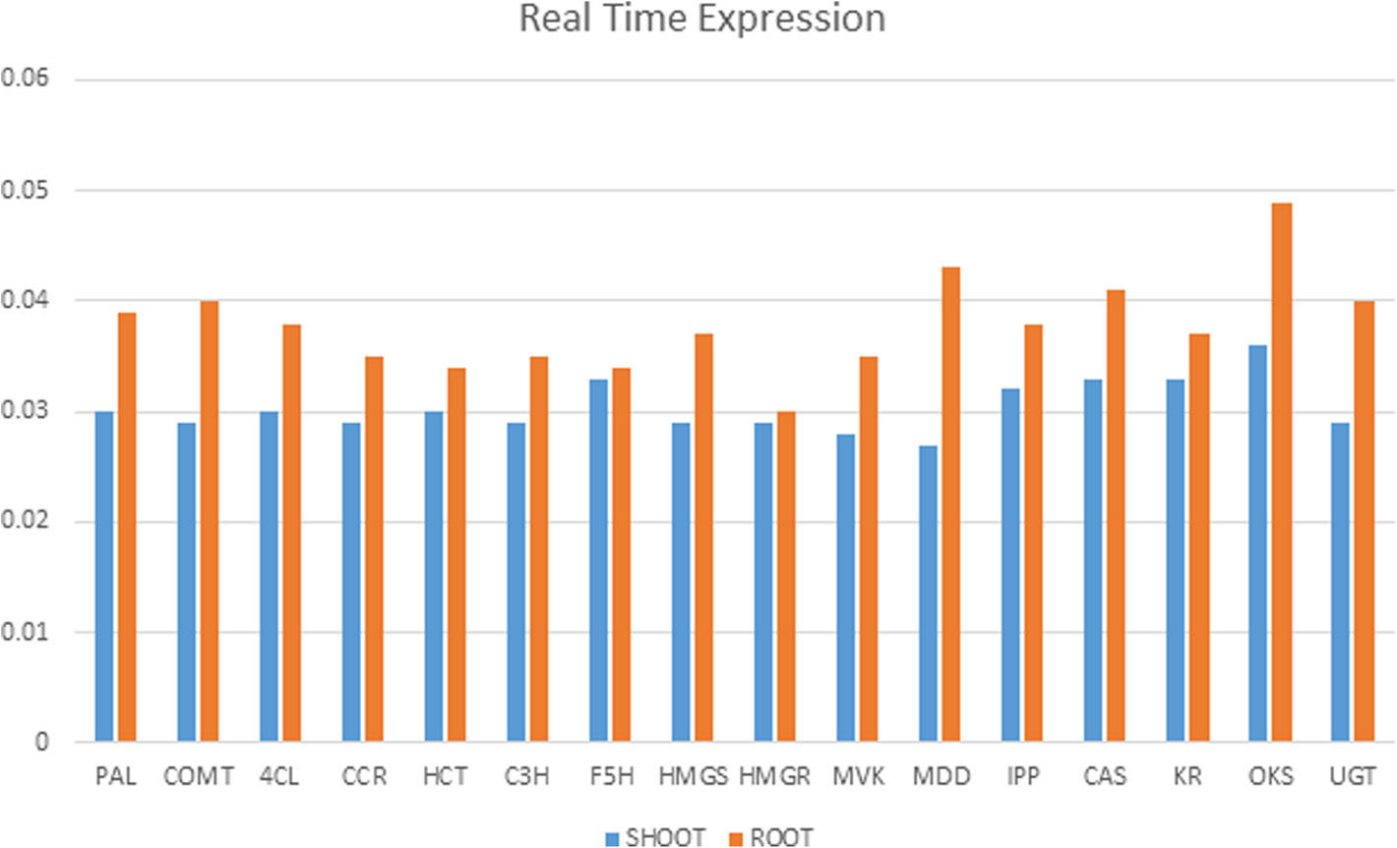
Real time expression bar diagram for root vs. shoot for the selected 16 unigenes involved in lignin, saponin and anthraquinone biosynthesis pathway in *Aloe vera* which represents the higher expression of these unigenes in root as compared to shoot

Differential expression of unigenes identified in root and leaf was assayed by calculating FPKM (Fragments per kilobase of transcript per million mapped reads) values obtained from aligning the root and leaf high quality reads against a reference transcriptome formed by clustering both the samples unigenes. Transcripts were further classified as up and down regulated based on their log fold change (FC) value calculated by FC = Log2 (Treated/Control) formula. FC value greater than zero were considered up-regulated whereas less than zero were down-regulated. Threshold *P* value was taken 0.05 to filter statistically significant results. A total of 59,188 CDS were found commonly expressed both in root and leaf tissue while 1427 genes were significantly up-regulated and 2208 genes were found down-regulated in leaf tissue as compared to root according to differential gene expression values. A heat map was constructed using the log-transformed and normalized values of genes based on Pearson correlation distances as well as based on complete linkage method. (Additional file 2: Heat map of differentially expressed genes Leaf vs Root). A separate heat map was also generated for the identified unigenes that encode for different enzymes involved in secondary metabolites biosynthesis in root and leaf tissue (Fig. 12). Genes encoding saponin biosynthesis like acetyl-CoA acetyltransferase (ACT), mevalonate kinase (MVK), phosphomevalonate kinase (PMVK), mevalonate-5-diphosphate decarboxylase (MDD) and cycloartenol synthase (CAS) were up-regulated in root tissue while DOXP synthase and DOXP reductoisomerase genes were found to be up-regulated in leaf tissue. UDP glycosyltransferase (UGT) and octaketide synthase genes (OKS) were highly expressed in both root and leaf tissue. Quantitative real-time PCR assay, as Ct values, was performed for the 16 putative genes belonging to lignin, saponin and aloin biosynthesis pathway. The unigenes related to saponin pathway i.e. HMG-CoA reductase (root CDS_10956_ Unigene_36039; leaf CDS_6769_Unigene_ 31,011), mevalonate kinase (root CDS_20142_ Unigene_50309; leaf CDS_25786_Unigene_64544), mevalonate-5 diphosphate decarboxylase (root CDS_24864_Unigene_57650; leaf CDS_29708_Unigene_71225), isopentenyl-PP isomerase (root CDS_24864_Unigene_57650; leaf CDS_25506_ Unigene_64122) and cycloartenol synthase (root CDS_16290_Unigene_44550; leaf CD_11153_ Unigene_41455) were expressed at higher level in root than in leaf tissue as given in DGE expression data. HMG-CoA synthase (root CDS_12664_Unigene_38987; leaf CDS _14177_ Unigene _46706) showed higher expression in root tissue than leaf. Enzymes related to aloin biosynthesis i.e. keto reductase (root CDS_22866_ Unigene_54394; leaf CDS_15459_Unigene_48740), octaketide synthase (root CDS_ 8049 _Unigene_30214; leaf CDS_36399_ Unigene_84377), UDP-Glycosyltransferase (root CDS_5315_Unigene_22956; leaf CDS_1908_Unigene_10,506), were highly expressed in both root and leaf tissue with somewhat higher expression in root than leaf. Genes encoding lignin biosynthesis pathway were also expressed at higher level in *Aloe vera* root as compared to leaf tissue as presented in DGE data (Fig. 13) (Tables 6, 7 and 8).

**Table 6:** Representing fold change in gene expression after 6,12and 24 h of methyl jasmonate treatment

Pathway name	Gene Name	Fold Change after 6 h of methyl jasmonate treatment	Fold Change after 12 h methyl jasmonate treatment	Fold Change after 24 h methyl jasmonate treatment
Saponin biosynthesis Pathway	HMGS	5.74	4.86	222.86
	HMGR	491.14	5042.76	16.56
	MVK	250.73	1910.85	364.55
	MDD	6653.97	18,820.27	7643.41
	IPP	16.8	44.33	3.36
	CAS	0.76	1.06	2.89
Anthraquinone biosynthesis pathway	KR	4.99	9.06	16.79
	OKS	2.75	1.02	1910.85
	UGT	4.72	6.91	20.97
Lignin biosynthesis pathway	PAL	1.34	1.09	0.2
	COMT	344.89	7.67	14.03
	4CL	22.16	124.49	70.52
	CCR	8	3.2	1.67
	HCT	0.71	1.66	0.57

**Table 7:** Candidate genes related to secondary metabolism identified in root by KEGG Pathway Mapping

Pathway	Enzyme Name	EC Number	Root Gene ID	Sequence Length	E value
Lignin Pathway	Phenylalanine ammonia lyase	EC:4.3.1.24	CDS_26921_Unigene_60717	2142	0
	Caffeoyl CoA O-methyltransferase	EC:2.1.1.104	CDS_5516_Unigene_23585	720	2.69E-122
	Caffeic acid O-methyltransferase	EC:2.1.1.68	CDS_33315_Unigene_71353	1092	0
	4-Coumarate:coenzyme A(CoA) ligase	EC:6.2.1.12	CDS_14099_Unigene_41223	1830	4.56E-164
	Cinnamoyl-CoA reductase	EC:1.2.1.44	CDS_7112_Unigene_27968	1110	0
	Hydroxycinnamoyl transferase	EC:2.3.1.133	CDS_33828_Unigene_72226	1308	0E + 00
	Cinnamate-4-hydroxylase	EC:1.14.13.11	CDS_10076_Unigene_34389	1584	0
	4-Coumarate 3-hydroxylase	EC:1.14.13.36	CDS_7933_Unigene_29870	1668	0.00E + 00
	Ferulate 5-hydroxylase	EC: 1.14.-.-	CDS_18772_Unigene_48194	1554	0
	Cinnamyl alcohol dehydrogenase	EC:1.1.1.195	CDS_10431_Unigene_35075	1065	0
Saponin Pathway	acetyl-CoA C-acetyltransferase	EC:2.3.1.9	CDS_18125_Unigene_47323	1248	0
	HMG-CoA synthase	EC:2.3.3.10	CDS_12664_Unigene_38987	1407	0
	HMG-CoA reductase	EC:1.1.1.34	CDS_10956_Unigene_36039	1728	0
	Mevalonate kinase	EC:2.7.1.36	CDS_20142_Unigene_50309	1167	0
	Phosphomevalonate kinase	EC:2.7.4.2	CDS_24841_Unigene_57598	1551	0
	Mevalonate-5-diphosphate decarboxylase	EC:4.1.1.33	CDS_26736_Unigene_60412	1266	0
	Isopentenyl-PP isomerase	EC:5.3.3.2	CDS_24864_Unigene_57650	1071	0
	Farnesyl diphosphate synthase	EC:2.5.1.1 2.5.1.10	CDS_4879_Unigene_21816	1119	0
	Squalene synthase	EC:2.5.1.21	CDS_3000_Unigene_14589	1230	0
	Squalene epoxidase	EC:1.14.14.17	CDS_7723_Unigene_29373	1329	0
	Cycloartenol synthase	EC:5.4.99.8	CDS_16290_Unigene_44550	2463	0
	DOX Phosphate synthase	EC:2.2.1.7	CDS_29944_Unigene_65832	2163	0
	DOXP Reductoisomerase	EC:1.1.1.267	CDS_3999_Unigene_18493	1413	0
Aloin Pathway	keto reductase	EC:1.1.1.184	CDS_22866_Unigene_54394	1164	0
	Octaketide synthase	EC:2.3.1.-	CDS_8049_Unigene_30214	1212	5.63E-76
	UDP glycosyltransferase	EC:2.4.1.-	CDS_5315_Unigene_22956	2112	1.14E-62
Carotenoid Pathway	phytoene synthase	EC:2.5.1.32	CDS_17552_Unigene_46473	1251	0
	15-cis-phytoene desaturase	EC:1.3.5.5	CDS_15090_Unigene_42743	918	0
	zeta-carotene isomerase	EC:5.2.1.12	CDS_23145_Unigene_54818	1140	0
	prolycopene isomerase	EC:5.2.1.13	CDS_13175_Unigene_39706	1070	0
	lycopene beta-cyclase	EC:5.5.1.19	CDS_12118_Unigene_38053	1533	0
	lycopene epsilon-cyclase	EC:5.5.1.18	CDS_14946_Unigene_42534	1707	0
	zeaxanthin epoxidase	EC:1.14.13.90	CDS_28676_Unigene_63548	1992	0
	violaxanthin de-epoxidase	EC:1.23.5.1	CDS_37413_Unigene_78465	1404	0
	abscisic-aldehyde oxidase	EC:1.2.3.14	CDS_12581_Unigene_38831	4152	0

**Table 8:** Candidate genes related to secondary metabolism identified in leaf by KEGG Pathway Mapping

Pathway	Enzyme Name	EC Number	Leaf Gene ID	E value	Sequence length
Lignin pathway	PAL:Phenylalanine ammonia-lyase	EC:4.3.1.24	CDS_25139_Unigene_63439	0	2142
	CCoAOMT: Caffeoyl CoA O-methyltransferase	EC:2.1.1.104	CDS_8759_Unigene_36297	1.02E-121	807
	COMT: Caffeic acid O-methyltransferase	EC:2.1.1.68	CDS_32183_Unigene_76237	0	1092
	4CL: 4-Coumarate:coenzyme A(CoA) ligase	EC:6.2.1.12	CDS_30808_Unigene_73318	0	1662
	CCR: Cinnamoyl-CoA reductase	EC:1.2.1.44	CDS_30855_Unigene_73445	7.34E-146	1017
	HCT: Hydroxycinnamoyl transferase	EC:2.3.1.133	CDS_18828_Unigene_54046	6.33E-127	777
	C4H: Cinnamate-4-hydroxylase	EC:1.14.13.11	CDS_13347_Unigene_45300	0	1653
	C3H: 4-Coumarate 3-hydroxylase	EC:1.14.13.36	CDS_6947_Unigene_31430	0	1593
	F5H: Ferulate 5-hydroxylase	EC:1.14.-.-	CDS_13705_Unigene_45900	2.09E-167	1083
	CAD: Cinnamyl alcohol dehydrogenase	EC:1.1.1.195	CDS_12114_Unigene_43205	0	1062
Saponin pathway	Acetyl-CoA acetyltransferase	EC:2.3.1.9	CDS_15891_Unigene_49443	0	1248
	HMG-CoA synthase	EC:2.3.3.10	CDS_14177_Unigene_46706	0	1407
	HMG-CoA reductase	EC:1.1.1.34	CDS_6769_Unigene_31,011	0	1749
	Mevalonate kinase	EC:2.7.1.36	CDS_25786_Unigene_64544	0	1167
	Phosphomevalonate kinase	EC:2.7.4.2	CDS_25395_Unigene_63890	0	1551
	Mevalonate-5-diphosphate decarboxylase	EC:4.1.1.33	CDS_29708_Unigene_71225	0	1266
	Isopentenyl-PP isomerase	EC:5.3.3.2	CDS_25506_Unigene_64122	2.72E-154	1161
	Farnesyl diphosphate synthase	EC:2.5.1.1 2.5.1.10	CDS_11635_Unigene_42360	0	1221
	Squalene synthase	EC:2.5.1.21	CDS_463_Unigene_2687	0	1230
	Squalene epoxidase	EC:1.14.14.17	CDS_5257_Unigene_26107	0	1575
	Cycloartenol synthase	EC:5.4.99.8	CDS_11153_Unigene_41455	0	2457
	DOXP(1-deoxy-D-xylulose-5-phosphate)synthase	EC:2.2.1.7	CDS_22770_Unigene_59944	0	2223
	DOXP(1-deoxy-D-xylulose-5-phosphate) reductoisomerase	EC:1.1.1.267	CDS_20571_Unigene_56673	0	1413
Anthroquinone Pathway	Keto reductase	EC:1.1.1.184	CDS_15459_Unigene_48740	0	1299
	Oktaketide synthase	EC:2.3.1.-	CDS_36399_Unigene_84377	1.23E-109	1212
	UDP Glycosyltransferase	EC:2.4.1.-	CDS_1908_Unigene_10,506	1.95E-35	2112
Carotenoid pathway	phytoene synthase	EC:2.5.1.32	CDS_7115_Unigene_31975	0	1197
	15-cis-phytoene desaturase	EC:1.3.5.5	CDS_13567_Unigene_45668	0	1710
	zeta-carotene isomerase	EC:5.2.1.12	CDS_30973_Unigene_73723	0	1083
	prolycopene isomerase	EC:5.2.1.13	CDS_11458_Unigene_41985	0	1776
	lycopene beta-cyclase	EC:5.5.1.19	CDS_7694_Unigene_33583	0	1104
	lycopene epsilon-cyclase	EC:5.5.1.18	CDS_22879_Unigene_60075	0	1620
	zeaxanthin epoxidase	EC:1.14.13.90	CDS_25413_Unigene_63924	0	1992
	violaxanthin de-epoxidase	EC:1.23.5.1	CDS_22464_Unigene_59446	0	1401
	abscisic-aldehyde oxidase	EC:1.2.3.14	CDS_21911_Unigene_58585		4152

As the secondary metabolites are considered to be produced as a defense mechanism of plants under various biotic and abiotic stresses, [43] here stress condition was provided to the plant by spraying a chemical elicitor, methyl jasmonate at time interval of 6, 12 and 24 h to stimulate the gene expression. Relative expression of genes using real-time quantitative PCR were calculated with 2− ΔΔCT method as described by Livak and Schmittgen, 2001 [44] [Here ΔΔCT = (*C*T_target_ -*C*T_GAPDH_) time *x* - (*C*T_target_ - *C*T_GAPDH_) time 0].

**Fig. 14:**
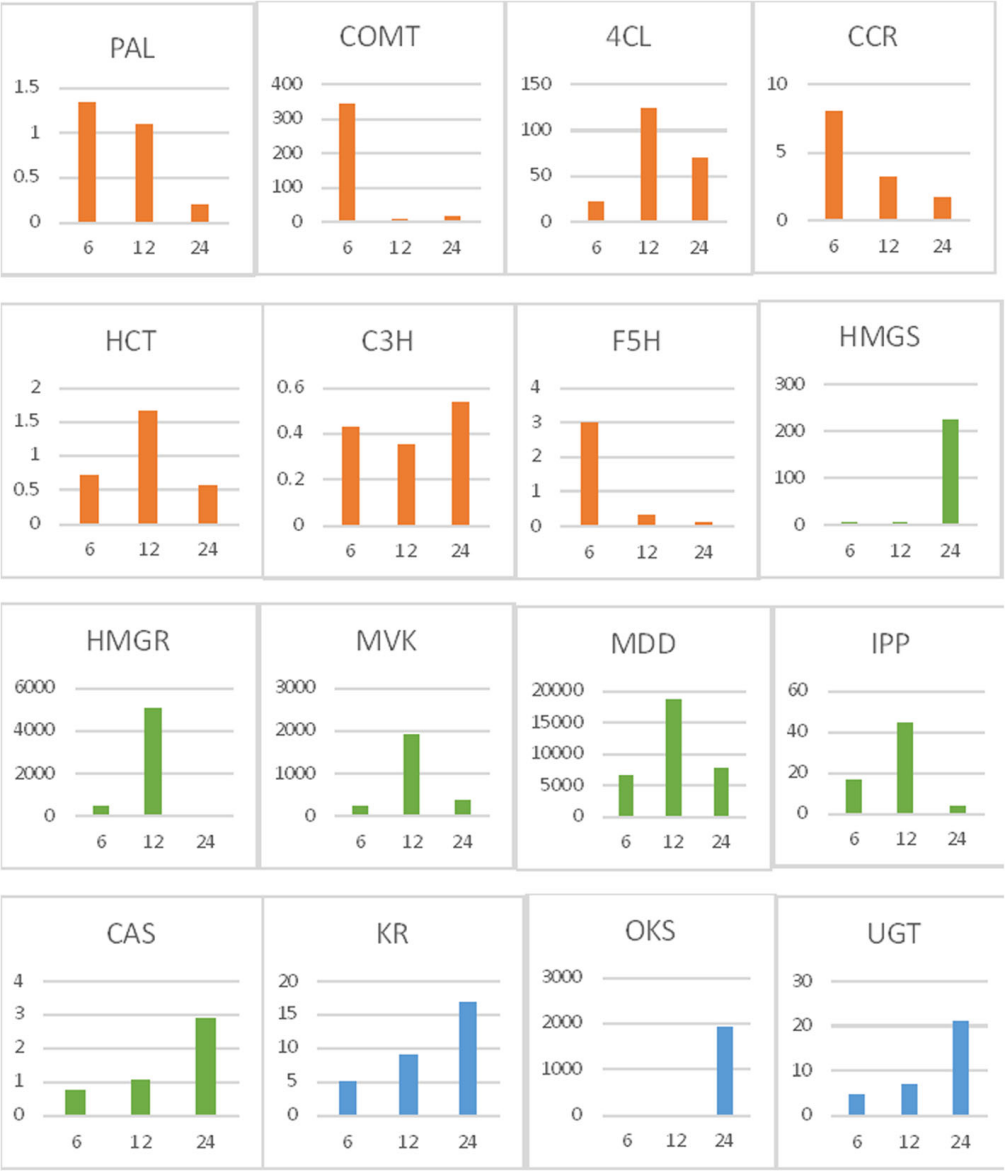
Methyl Jasmonate induced expression in leaf after 6, 12 and 24 h. Different colours representing the unigenes of different pathways: orange coloured are the unigenes of lignin pathway green coloured are the unigenes of saponin pathway and blue coloured are the unigenes of anthraquinone pathway. Vertical axis representing fold change of the unigenes with time

Maximum gene expression was found after 12 h of treatment in HMGR (CDS_6769_Unigene_ 31,011,~ 5042.76 fold), MVK (CDS_25786_Unigene_64544, ~ 1910.85 fold), MDD (CDS_29708_Unigene_71225, ~ 18,820.27 fold) and IPP isomerase (CDS_25506_Unigene_64122, ~ 44.33 fold) and decline in the expression was noticed after 24 h of induction. Interestingly, HMGS (CDS _14177_ Unigene _46706, ~ 200 fold) and CAS (CD_11153_Unigene_41455, ~ 3 fold) were highly expressed after 24 h of treatment. Unigenes of aloin biosynthesis pathway were also upregulated and showed maximum expression after 24 h of methyl jasmonate induction (KR, CDS_15459_Unigene_48740, ~ 16.8 fold; OKS, CDS_36399_ Unigene_84377, ~ 1910.8 fold; UGT, CDS_1908_Unigene_ 10,506, ~ 20.9 fold). Putative genes of lignin pathway PAL (CDS_25139_Unigene_63439), COMT (CDS_32183_Unigene_76237), 4CL (CDS_30808_Unigene_73318), CCR (CDS_30855_Unigene_73445), HCT (CDS_18828_Unigene_54046), C3H (CDS_6947_Unigene_31430) and F5H (CDS_13705_Unigene_45900) were also analyzed for relative expression after methyl jasmonate induction and an increase in fold change was noticed after 6 h of treatment while many of the genes were down-regulated after 12 and 24 h of MeJa treatment (Fig. 14).

## Discussion

*Aloe vera* is one of the most popular medicinal plant used worldwide nevertheless, hardly any work has been conducted on its functional genomics. Only a few nucleotide sequences encoding complete or partial gene sequences are available in public databases like NCBI. No ESTs or genome survey sequences (GSS) from *Aloe vera* have been deposited in the GenBank [45]. Therefore, results of this investigation on whole transcriptome sequencing of *Aloe vera* root as well as leaf tissues are important in the perspective of functional genomics of the plant.

### *De novo* transcriptome assembly and functional annotation

A number of high quality reads, fine transcripts and CDS length obtained from *Aloe vera* next generation transcriptome sequencing indicates the functionality of *Aloe vera s*equencing at first sight. CDS were functionally annotated and most of the identified CDS resulted into the significant blast hits for root and leaf samples respectively, which represented a big coverage of *Aloe vera* genome. The majority of hits were found to be against *Elaeis guineensis* followed by *Phoenix dactylifera* for both the samples, showing closer relationship with *Aloe vera* genotypically.

GO mapping was carried out to assign the functions of identified CDS and grouped into three main domains: Biological processes, molecular function and cellular component. From functional GO distribution it was concluded that in the biological processes maximum 5890 genes in root and 6329 genes in leaf were only limited to metabolism and even majority of top BLAST hits i.e. 980 hits in root sample and 922 in leaf sample were found to be involved in metabolic processes, proves high metabolic activity of our research plant.

### Transcription factor analysis

The transcription factors (TFs) are sequence specific DNA binding proteins that interact with the promoter regions of target genes to modulate their expression. TFs play a significant role in the regulation of plant development, reproduction, intercellular signaling, cell cycle, response to the environment as well as in modulation of secondary metabolite biosynthesis. Transcription factors like AP2/ERF, bHLH and MYB and NAC were found to be involved in regulating secondary metabolism [46, 47]. TFs family WRKYs have eminent role in regulating secondary metabolism during stress conditions [48]. Identified MYB and NAC transcriptional factors may function as switches in lignin biosynthesis pathways as observed in other plants [49]. Transcription factors identified in *Aloe vera* database may play a crucial role in modifying the levels of secondary metabolites, to stimulate the production of valuable secondary metabolites and to reduce the level of an undesirable metabolite that have an adverse effect on the quality of *Aloe vera.*

### KEGG pathway mapping and potential gene identification related to secondary metabolism

KEGG is the most widely used biological database in the world for biological interpretation of genome sequences using server KASS [50, 51]. The identified CDS were mapped to reference canonical pathways in KEGG and predicted genes were found potentially involved in 24 different KEGG pathway. Five different functional categories were allotted to isolated CDS which encompass metabolism, genetic information processing, environmental information processing, cellular processes and organismal systems. It was found that 194 genes in root and 192 genes in leaf were related to environmental adaptation which proves well adapted feature of *Aloe vera* in diverse conditions.

The major secondary metabolite related constituents of *Aloe vera* include anthraquinones, saponin, lignin, sterols, polysaccharides, alkylbenzenes, dehydrabietic acid derivatives, lactin and salicylic acid [52, 53] which attribute for its pharmacological activity. Biosynthetic pathway of secondary metabolites in *Aloe vera* remains undiscovered till date. Several putative genes related to saponin, lignin, anthraquinone, carotenoid and phenylpropanoid biosynthesis have been identified from *Aloe vera* root and leaf tissue by KEGG Pathway functional annotation. Saponins are the major secondary metabolites of *Aloe vera*, well known for pharmaceutical and cosmetic properties [54]. Saponins are a complex and chemically varied group of compounds consisting of triterpenoid or steroidal aglycones linked to oligosaccharide moieties. Steroidal saponins are hypothesized to share a common route with triterpene saponin from C5 isoprenoids, isopentenyl diphosphate (IPP) to the formation of the C30 unit squalene and 2, 3 oxidosqualene. The cytosolic MVA pathway was accepted as the only biosynthetic route to IPP until the plastid bound MEP pathway was elucidated in bacteria and plants. Now, it is generally suggested that sesquiterpenes, triterpenes and steroid are preferentially formed via MVA pathway, whereas, monoterpenes, diterpenes, and carotenoid are formed predominantly via the MEP pathway [55] There is no information as of today that which of the pathways (MVA or MEP) or both are involved in the biosynthesis of saponins precursors and subsequently the number of genes that are involved in the final biosynthesis of saponins *in planta* / *Aloe vera.* Squalene synthase (SQS) catalyzes the condensation of two farnesyl pyrophosphate (C15 unit) to 30 carbons compound squalene, the first committed intermediate in sterol and triterpene saponin biosynthesis pathway [56]. Squalene synthase is well characterized in various plants like *Chlorophytum borivilianum,* [57] *Dioscorea zingiberensis,* [58] and *Siraitia grosvenorii* [56]. Squalene epoxidase (SQE) with the cofactors O_2_ and NADPH catalyzes the conversion of squalene to 2,3-oxidosqualene that acts as a substrate for various oxidosqualene cyclases [59]. Oxidosqualene cyclases (OSC) catalyze the cyclization of 2, 3-oxidosqualene which is a branching point for the sterol and triterpenoids saponin synthesis [60]. Different OSCs have been characterized in the past few years and named after their respective products like lanosterol synthases,[61] cycloartenol synthases, [62] lupeol synthases [63] and β-amyrin synthases (BASs) [64, 65]. Glycosylation is the final step in steroidal saponin biosynthesis, regulating the biological activities of saponins, catalyzed by glycosyltransferases [66]. Several unigenes encoding acetyl-CoA acetyltransferase, HMG-CoA synthase, HMG-CoA reductase, mevalonate kinase, phosphomevalonate kinase, mevalonate-5-diphosphate decarboxylase, isopentenyl-PP isomerase, farnesyl diphosphate synthase, squalene synthase, squalene epoxidase, cycloartenol synthase, 1-deoxy-D-xylulose-5-phosphate synthase, 1-deoxy-D-xylulose-5-phosphate reductoisomerase and UDP glycosyltransferases which may be potentially involved in saponin biosynthesis pathway, have been identified from transcriptome sequencing. From *Aloe vera* transcriptome sequencing data no unigene encoding β-amyrin synthase was found, it denotes the lack of triterpenoid saponin in our research plant. However downward steps towards saponin biosynthesis are still unknown for *Aloe vera*.

Anthraquinones, another important group of secondary metabolites in *Aloe vera* are tricyclic aromatic quinines having strong antibacterial, antiviral, antifungal activity. Aloin, an anthraquinone glycoside is one of the most active metabolite of this group present in *Aloe vera* [67]. The conjugation of a metabolite with a sugar moiety eases its entry into the target cells leading to the enhancement of the pharmacological activity [68, 69]. To date, very little is known about the biosynthetic steps leading to the formation of aloin/anthraquinones in *Aloe vera*. Abe et al. [70] have identified from *Aloe arborescens* a plant-specific polyketide synthase of type-III called octaketide synthase (OKS), that shares 50% amino acid sequence identity with other plant enzymes belonging to chalcone synthase superfamily. OKS catalyzes the iterative condensation of eight molecules of malonyl-CoA, might be involved in the biosynthesis of the octaketide anthrone aloin in *Aloe vera*. However, recombinant OKS expressed in *E. coli* has been reported to produce unnatural octaketide SEK4 / SEK4b as derailed shunt products either due to misfolding or glycosylation of heterologously expressed recombinant protein or the absence of interactions with an unidentified tailoring enzyme possibly ketoreductases. The physiological role of OKS *in planta* is yet to be identified. Earlier Grun and Franz [71] studied the in vitro biosynthesis of aloin from aloe-emodin anthrone and reported that the enzyme responsible for the C- glycosylation of aloe-emodin anthrone is specific for UDP-Glc. The transfer of activated sugar like UDP-glucose to aglycone acceptor molecule is catalyzed by the enzyme UDP-glycosyltransferase. Various genes like octaketide/polyketide synthase, aldoketoreductase, and UDP-glycosyltransferase have been identified from transcriptome sequencing which can be useful for unravelling the aloin biosynthesis in *Aloe vera*.

Enzymes related to lignin biosynthesis viz. L-phenylalanine ammonia-lyase (PAL), caffeoyl CoA O-methyltransferase (CCoAOMT), caffeic acid O-methyltransferase (COMT), 4-coumarate:coenzyme A(CoA) ligase (4CL), cinnamoyl-CoA reductase (CCR), hydroxycinnamoyl transferase (HCT), cinnamate-4-hydroxylase (C4H), 4-coumarate 3-hydroxylase (C3H), ferulate 5-hydroxylase (F5H), cinnamyl alcohol dehydrogenase (CAD), meticulously investigated for their roles in plant development,[49] have been identified from the *Aloe vera* sequencing database which may be helpful to reveal the steps toward the lignin biosynthesis in *Aloe vera*.

*Aloe vera* has been reported to have anti-aging effect similar to vitamin A derivatives. Carotenoids in *Aloe vera*, serve as precursors of vitamin A in human diet, and are of interest as potential anti-cancer agents [72]. In the past few years, genes encoding enzymes involved in carotenoid biosynthesis in plants have been identified and characterized at the molecular level. Briefly, geranylgeranyl diphosphate (GGPP) which acts as a precursor for carotenoids is synthesized by recruiting MEP pathway of isoprenogenesis. Further, condensation of two GGPP molecules is catalyzed by phytoene synthase (PSY). Subsequent desaturation by phytoene desaturase produces lycopene while the next tier of modifications is catalyzed by cyclases, hydroxylases, and ketolases, resulting in the production of different carotenoids [73]. Various genes belonging to carotenoid biosynthesis including phytoene synthase, 15-cis-phytoene desaturase, zeta-carotene isomerase, prolycopene isomerase, lycopene beta-cyclase, lycopene epsilon-cyclase, zeaxanthin epoxidase, violaxanthin de-epoxidaseand abscisic-aldehyde oxidase have been isolated from sequencing, may pave the way of carotenoid biosynthesis in *Aloe vera.* In short, the unigenes related to different secondary metabolites biosynthesis, identified from transcriptome sequencing may unravel the secondary metabolism which is most responsible for potent applications of *Aloe vera* and still undiscovered.

### Differential gene expression analysis and real-time expression analysis of transcripts involved in secondary metabolism

Differential Gene Expression **(**DGE) enables quick and thorough analysis of the gene expression under various conditions for a variety of tissues as a comparative landscape [74]. Based on FPKM values, it was revealed that most of the genes related to secondary metabolism were highly expressed in root as compared to leaf tissue. Most of the root specific transcripts for saponin biosynthesis pathway were found up regulated in DGE data, same was also reported in *Asparagus racemosus* for steroidal saponin biosynthesis [75].

Several putative genes involved in saponin biosynthesis like acetyl-CoA acetyltransferase (ACT), mevalonate kinase (MVK), phosphomevalonate kinase (PMVK), mevalonate-5-diphosphate decarboxylase (MDD) and cycloartenol synthase (CAS) were up-regulated in root tissue while DOXP synthase and DOXP reductoisomerase genes were found to be up-regulated in leaf tissue indicating that plastid bound MEP pathway for saponin biosynthesis is dominant in leaf tissue. UDP glycosyltransferase (UGT) and octaketide synthase genes (OKS) were highly expressed in both root and leaf tissue stipulate that aloin is synthesized in both root and leaf tissue in *Aloe vera*.

The results obtained from quantitative real-time PCR assay of selected genes of saponin biosynthesis including HMG-CoA synthase, HMG-CoA reductase, mevalonate kinase, mevalonate-5 diphosphate decarboxylase, isopentenyl-PP isomerase and cycloartenol synthase reveal that mostly genes were found more expressed in root than in leaf tissue as given in DGE expression data. Exceptionally, HMG-CoA synthase showed higher expression in root tissue which was revert to DGE expression value. From quantitative real time expression results it was UDP-glycosyltransferase, found highly expressed in both root and leaf tissue with somewhat more expression in root indicating biosynthesis of anthraquinones in leaf as well as root. Putative genes encoding lignin biosynthesis pathway were also substantially better expressed in *Aloe vera* root as compared to leaf tissue as presented in DGE data indicating its reliability.

The secondary metabolites are considered to be produced as a defense mechanism of plants against various undesirable environmental encounters including biotic and abiotic stresses [43]. Several studies have demonstrated that chemical elicitors like methyl jasmonate mediate such metabolic responses to environment through an extensive transcriptional reprogramming of the plant metabolism. In this study it was observed that most of the genes of saponin pathway were up-regulated on exposure of leaf tissue to methyl jasmonate, similar to that reported in *Asparagus racemosus*, a saponin rich medicinal plant [75]. Expression of HMG-CoA reductase, mevalonate kinase, mevalonate-5 diphosphate decarboxylase and isopentenyl-PP isomerase genes was maximum after 12 h of treatment and declined after 24 h of treatment while HMG-CoA synthase and cycloartenol synthase showed maximum expression after 24 h of treatment. The aglycone moiety of saponins is a triterpene derivative. Triterpenes are usually synthesized predominantly via mevalonate pathway of isoprenogenesis wherein HMGS catalyzes a step that not only holds the second degree of regulatory position but also produces a precursor for the primary regulatory step, the HMGR catalyzed reaction. The observed high expression of CAS is in alignment with the elevation in saponin biosynthesis through improved production of triterpene alcohols in preference to triterpene hydrocarbon like β-amyrin through carbon flux control at this branch point in favour of sterols. Failure to have a detectable level of expression of β-amyrin synthase is an avowal of this postulation. Aloin biosynthesis pathway related genes were also upregulated and showed maximum expression after 24 h of methyl jasmonate induction, indicating the correlation of both aloin and saponin with defense mechanism of *Aloe vera*. There was a little fold change in expression was noticed for the putative genes encoding lignin biosynthesis, showing no significant role of lignin in plant defense mechanism during stress conditions.

*De novo* assembly of transcriptome data in conjugation with DGE analysis served as a powerful approach for the identification of genes involved in the biosynthesis of important secondary metabolites pertaining to different chemical classes in *Aloe vera* - a non-model plant. The transcriptome database generated by this study will provide an important resource that may aid in identification and characterization of gene related to the specialized metabolism in the plant as well as understanding the function of gene set(s) in the biology and physiology of plant, metabolic pathways and their regulations, signal transduction mechanism, and marker-assisted breeding particularly for chemotype development in this species as well as other species of genus *Aloe*.

## Conclusions

*Aloe vera* is well known plant used worldwide for its medicinal and cosmetic properties due to its specialized metabolic competence. However, despite significant knowledge on chemical composition and healthful properties, any significant information about its genomics is completely lacking. Therefore, in the present study, transcriptome sequence data for *Aloe vera* root and shoot was generated using NGS technology. The transcriptome sequences have been assembled, annotated and analyzed with special emphasis on secondary metabolism. The assembly and genes have been validated by gene expression analysis. The potential genes isolated can be exploited for characterization of metabolic understanding and modulation of saponin, aloin, carotenoid and lignin biosynthesis in *Aloe vera*. Identified transcription factors may be recruited to understand their relative regulatory significance across different metabolic processes in the plant and undertake metabolic engineering studies. To our knowledge, this would be the first transcriptome sequencing study of *Aloe vera* until now.

## Methods

### Sample preparation and Total RNA isolation

*Aloe vera* was grown in the herbal nursery at Guru Jambheshwar University of Science and Technology, Hisar, India. Young leaves and roots were collected from the healthy plant, snap freezed in liquid nitrogen and stored at − 80 °C for further use. Total RNA was isolated from each tissue using RNeasy Plant Mini Kit (Qiagen) according to manufacturer’s instructions. The quality of the isolated RNA was checked on 1% denaturing agarose gel for the presence of 28S and 18S bands. Further, the RNA quality and quantity was analysed by using Qubit fluorometer.

### Library preparation

Total RNA isolated from the plant samples was used for the preparation of RNA-Seq paired end sequencing libraries with the help of TrueSeq® Stranded mRNA sample preparation kit (Illumina). Enrichment of mRNA from the total RNA was done with the help of poly-T attached magnetic beads which was followed by enzymatic fragmentation and 1st strand cDNA conversion. The second strand was then synthesised form the 1st strand using second strand mix and Act-D mix to facilitate RNA dependent synthesis. Then the double stranded cDNA samples were purified using AMPure XP beads (Agencourt Biosciences). These beads selectively binds larger double stranded cDNA samples and excess of primers, nucleotides, salts and enzymes were removed making the products free from any kind of contaminants. It was then followed by adapter ligation, A-tailing and enrichment by limited number of PCR cycles. The PCR amplified library was analyzed in Tape Station 4200 (Agilent Technologies) using High Sensitivity (HS) D5000 Screen Tape assay kit as per manufacturer instructions.

### Sequencing and quality control

The cDNA library was then used for paired end sequencing using Illumina Hi-Seq platform (2 × 150 bp chemistry) to generate the raw data for both the samples. In paired end sequencing the template fragment is sequenced in both forward and reverse directions. The samples were allowed to bind with complementary adapter oligos on paired-end flow cell with the help of kit reagents. The adapters were designed in order to allow selective cleavage of the forward strands after re-synthesis of the reverse strand during sequencing. The opposite end of the fragment was then sequenced from the copied reverse strand. Prior to the assembly the raw data obtained was processed to obtain high quality reads. Trimmomatic v0.35 was used to remove adapter sequences, ambiguous reads (reads with unknown nucleotides “N” larger than 5%), and low-quality sequences (reads with more than 10% quality threshold (QV) < 20 phred score). A minimum length of 100 nt (nucleotide) after trimming was applied. After removing the adapter and low quality sequences from the raw data high quality reads were retained for root and leaf sample respectively. This high quality (QV > 20), paired-end reads were used for *de-novo* assembly.

### *De-novo* transcriptome assembly, validation and CDS prediction

The high quality reads for both the samples were then assembled into transcripts using RNA-Seq assembler Trinity [41]. While assembling the transcripts there is a chance that large amounts of misassembled transcripts, erroneous and poorly supported transcripts may arise, therefore, all high quality reads were assembled with their respective assembled transcripts using Burrows-Wheeler Aligner [76]. The non-redundant transcripts were further clustered together using CD-HIT-EST-454 [77] at 95% identity and query coverage. After the assembly and clustering of transcripts, sequences were obtained that could not be extended further, these sequences were termed as unigenes. The unigenes were then used to predict coding sequences within them using TransDecoder.

### Gene ontology analysis

For the annotation, the predicted CDS were searched against NCBI non redundant(Nr) protein database (http://www.ncbi.nlm.nih.gov) using Basic local alignment search tool (BLASTx) with a common significance threshold cut-off of E-value ≤1e-05. Gene ontology (GO) annotations of the CDS were carried out with the help of Blast2GO program [78]. The BLASTx result accession IDs were searched directly in the gene product table (dbxref) of GO database. The GO mapping differentiated the predicted CDS into three major domains representing gene product properties namely: Biological process, Molecular function and Cellular component. Each predicted CDS may have more than one GO term assigned either in the same domain or in different domains i.e. biological process, molecular function and cellular component [79].

### Functional annotation of KEGG pathway

For the identification of possible involvement of the predicted CDS in various biological pathways, the CDS were mapped to the reference canonical pathways in Kyoto Encylopedia of Genes and Genomes (KEGG) database [80]. Five major divisions under which the CDS were distributed included metabolism, genetic information processing, environmental information processing, cellular processes and organismal systems. The information obtained upon KEGG analysis included KEGG Orthology (KO) assignments, their corresponding enzyme commission (EC) number and prediction of metabolic pathway using KEGG automated annotation server KASS [81].

### Abundance estimation and differential gene expression analysis (DGE)

FPKM (Fragments Per Kilobase of transcript per million mapped reads) values were calculated to measure the expression level of each assembled transcript sequence. For FPKM measurement a reference transcriptome was first generated by clustering both the samples unigenes i.e. leaf and root. The high-quality cleaned reads from each sample were aligned separately on reference transcriptome (clustered unigene of both the samples) using burrows wheeler aligner (bwa). The read count profile from the output file (.sam) of bwa alignment was generated by using SAMtools [82]. Differential gene expression (DGE) analysis was performed employing a negative binomial distribution model (DESeqv1.8.1 package http://www-huber.embl.de/users/anders/DESeq/) [83]. Dispersion values were calculated using following parameters: method = blind, sharing mode = fit-only and fit type = local. On the basis of log fold change (FC) the transcripts were further classified as up and down regulated. The log fold change value was calculated by using the formula: FC = Log2 (Treated/Control). Transcripts having FC value greater than zero were considered up-regulated whereas less than zero, were down-regulated. To obtain statistically significant results *P* value threshold of 0.05 was used. With the help of Multiple Experiment Viewer (MEV v4.9.0), a complete linkage hierarchical cluster analysis was performed on top 100 differentially expressed genes. A heat map (cluster) depicts the level of transcript abundance. Levels of expression are represented as log2 ratio of transcript abundance between leaf and root samples. A heat map was constructed employing the log-transformed and the normalized value of genes based on Pearson correlation distance as well as based on complete linkage method.

### Transcription factor analysis

The predicted CDS were searched against Plant transcription factor database (PlantTFdb) [84] to obtain the transcription factors from both root and leaf CDS.

### Gene validation with qRT-PCR

The transcripts obtained by sequencing were further validated by qRT-PCR. Sixteen unigenes involved in anthraquinone, saponin and lignin biosynthesis were selected for quantitative real-time expression. RNA was isolated with the help of CIA-PCIA method from the root and leaf samples as well as from the plant which was treated externally with methyl jasmonate (250 μM) at time interval of 6, 12 and 24 h. Isolated RNA was further reverse transcribed with the help of Revert Aid First Strand cDNA Synthesis Kit (Thermo Scientific) using oligo dT(18) primers. Specific primers were designed for sixteen unigenes and two housekeeping genes (GAPDH and beta tubulin) with the help of Primer Express software v3.0.1 (List of primers is given in Additional file 3). The qRT-PCR was carried out in triplicates using SYBR® Green Jump Start™ Taq Ready Mix™ (Sigma) on Applied Biosystems’ Step One™ Real Time PCR System. The reaction mixture used included 10 μl of SYBR green master mix, 20 pmol/μl forward and reverse primers and 2 μl of cDNA for a reaction volume of 20 μl. The thermal cycle used was as follows: initial denaturation at 95 °C for 20 s followed by denaturation and annealing at 95 °C for 3 s and 60 °C for 30 s for 40 cycles followed by melt curve analysis: 95 °C for 15 s, 60 °C for 1 min and 95 °C for 15 s. The relative expression levels were determined using 2^-∆∆Ct^ method [44].

## Additional files

Additional file 1: **Table S1** and **S2.**: Top 10 most represented GO terms of 3 major GO domain in root and leaf. (DOCX 15 kb)

Additional file 2: Heat map of differentially expressed genes Leaf vs Root**.** (PNG 507 kb)

Additional file 3: List of primers used for real time PCR. (DOCX 12 kb)
