# Supplementary material for: De novo sequencing, assembly and characterisation of Aloe vera transcriptome and analysis of expression profiles of genes related to saponin and anthraquinone metabolism
Source: BMC Genomics. 2018 Jun 1;19:427. doi: 10.1186/s12864-018-4819-2 (PMC5984767; doi:10.1186/s12864-018-4819-2)
Supplement: Supplementary file 1 — Table S1 and S2.: Top 10 most represented GO terms of 3 major GO domain in root and leaf. (DOCX 15 kb) [file 12864_2018_4819_MOESM1_ESM.docx]

| **Biological process** | | | **Cellular Component** | | | **Molecular Function** | | |
| --- | --- | --- | --- | --- | --- | --- | --- | --- |
| **GO Id** | **# Hits** | **GO Term** | **GO Id** | **# Hits** | **GO Term** | **GO Id** | **# Hits** | **GO Term** |
| **GO:0008152** | 980 | Metabolic process | **GO:0016021** | 2231 | Integral component of membrane | **GO:0005524** | 1189 | ATP binding |
| **GO:0055114** | 792 | Oxidation-reduction process | **GO:0016020** | 638 | Membrane | **GO:0046872** | 506 | Metal ion binding |
| **GO:0006468** | 382 | Protein phosphorylation | **GO:0005634** | 507 | Nucleus | **GO:0003676** | 424 | Nucleic acid binding |
| **GO:0006412** | 296 | Translation | **GO:0005737** | 371 | Cytoplasm | **GO:0008270** | 391 | Zinc ion binding |
| **GO:0006508** | 275 | Proteolysis | **GO:0009507** | 219 | Chloroplast | **GO:0016491** | 355 | Oxidoreductase activity |
| **GO:0055085** | 268 | Transmembrane transport | **GO:0005840** | 205 | Ribosome | **GO:0000166** | 315 | Nucleotide binding |
| **GO:0006355** | 214 | Regulation of transcription, DNA-templated | **GO:0005622** | 204 | Intracellular | **GO:0003677** | 308 | DNA binding |
| **GO:0005975** | 201 | Carbohydrate metabolic process | **GO:0005739** | 198 | Mitochondrion | **GO:0003735** | 302 | Structural constituent of ribosome |
| **GO:0006886** | 160 | Intracellular protein transport | **GO:0005829** | 192 | Cytosol | **GO:0004672** | 263 | Protein kinase activity |
| **GO:0016310** | 152 | Phosphorylation | **GO:0005886** | 170 | Plasma membrane | **GO:0005525** | 217 | GTP binding |

**Table 1: Top 10 most represented GO terms of 3 major GO domain in Root Sample**

**Table 2: Top 10 most represented GO terms of 3 major GO domain in Leaf Sample**

| **Biological Process** | | | **Cellular Component** | | | **Molecular Functions** | | |
| --- | --- | --- | --- | --- | --- | --- | --- | --- |
| **GO Id** | **# Hits** | **GO Term** | **GO Id** | **# Hits** | **GO Term** | **GO Id** | **# Hits** | **GO Term** |
| **GO:0008152** | 922 | Metabolic process | **GO:0016021** | 2330 | Integral component of membrane | **GO:0005524** | 1323 | ATP binding |
| **GO:0055114** | 820 | Oxidation-reduction process | **GO:0016020** | 574 | Membrane | **GO:0003676** | 604 | Nucleic acid binding |
| **GO:0006468** | 548 | Protein phosphorylation | **GO:0005634** | 510 | Nucleus | **GO:0046872** | 588 | Metal ion binding |
| **GO:0006412** | 282 | Translation | **GO:0005737** | 347 | Cytoplasm | **GO:0008270** | 530 | Zinc ion binding |
| **GO:0006508** | 256 | Proteolysis | **GO:0009507** | 271 | Chloroplast | **GO:0000166** | 355 | Nucleotide binding |
| **GO:0055085** | 250 | Transmembrane transport | **GO:0005739** | 207 | Mitochondrion | **GO:0004672** | 336 | Protein kinase activity |
| **GO:0005975** | 240 | Carbohydrate metabolic process | **GO:0005840** | 207 | Ribosome | **GO:0003677** | 325 | DNA binding |
| **GO:0015074** | 202 | DNA integration | **GO:0005622** | 205 | Intracellular | **GO:0016491** | 303 | Oxidoreductase activity |
| **GO:0006355** | 199 | Regulation of transcription, DNA-templated | **GO:0005886** | 205 | Plasma membrane | **GO:0003735** | 290 | Structural constituent of ribosome |
| **GO:0006886** | 158 | Intracellular protein transport | **GO:0005829** | 197 | Cytosol | **GO:0004674** | 282 | Protein serine/threonine kinase activity |
