# Supplementary figures and images for: De novo sequencing, assembly and characterisation of Aloe vera transcriptome and analysis of expression profiles of genes related to saponin and anthraquinone metabolism
Source: BMC Genomics. 2018 Jun 1;19:427. doi: 10.1186/s12864-018-4819-2 (PMC5984767; doi:10.1186/s12864-018-4819-2)

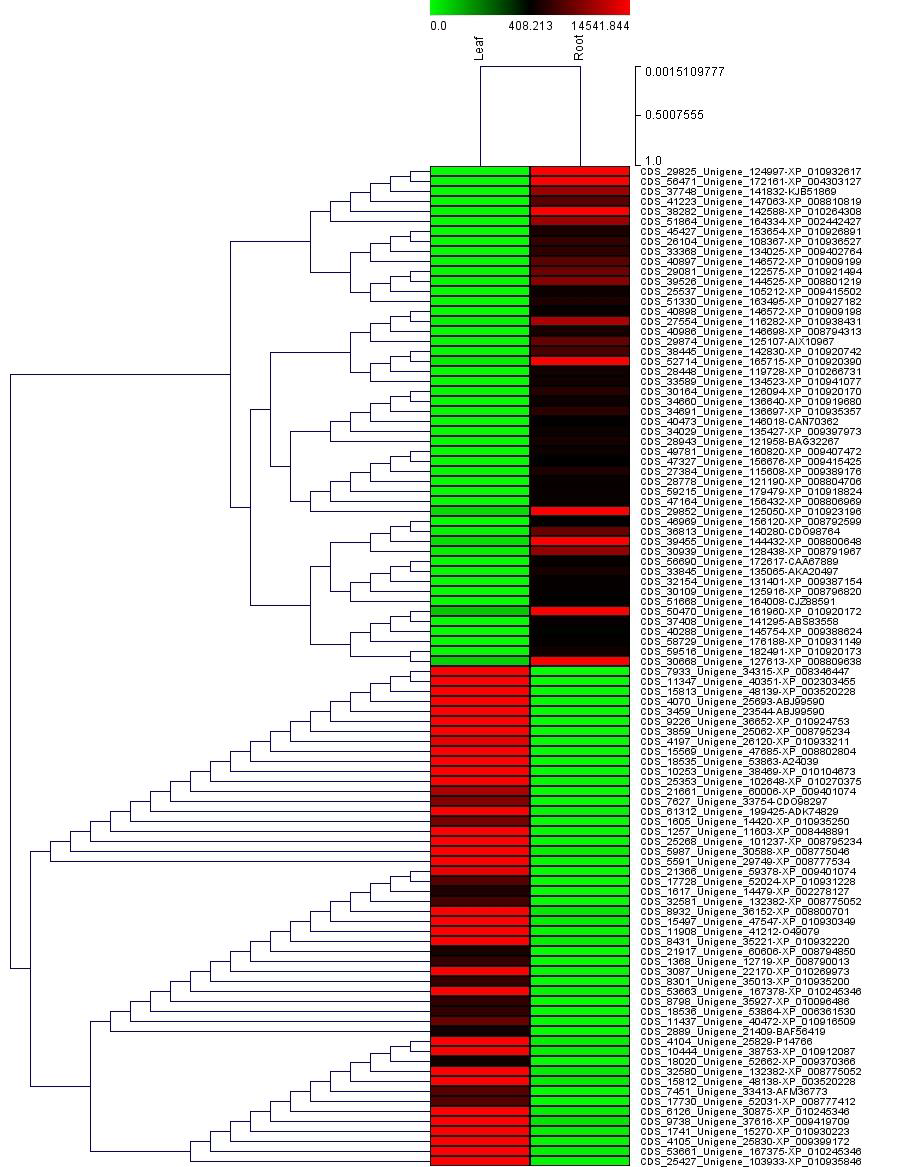

Supplement: Supplementary file 2 — Heat map of differentially expressed genes Leaf vs Root. (PNG 507 kb) [file 12864_2018_4819_MOESM2_ESM.png]
