# Supplementary material for: De novo sequencing, assembly and characterisation of Aloe vera transcriptome and analysis of expression profiles of genes related to saponin and anthraquinone metabolism
Source: BMC Genomics. 2018 Jun 1;19:427. doi: 10.1186/s12864-018-4819-2 (PMC5984767; doi:10.1186/s12864-018-4819-2)
Supplement: Supplementary file 3 — List of primers used for real time PCR. (DOCX 12 kb) [file 12864_2018_4819_MOESM3_ESM.docx]

| Primer name | Forward primer | Reverse Primer |
| --- | --- | --- |
| PAL | GCGAACCCGGTCACAAAC | TTAACGTCCTGGTTGTGTTGCT |
| COMT | TCAGGCTCAGGGCGTCAT | CCAGGGTTGTGAGCCAACA |
| 4CL | GATGGGAAGGGTGAAGAAGTATGA | CAGCGCCACAGCAAATCC |
| CCR | GCTTGAGGTCCGCAATTAGC | GTCGGACAAGCGGTATGGA |
| HCT | TCTATCGGCGGCCTGAAG | TCTTTCAACACCTCAGCATCGA |
| C3H | TGTGGAAGAACCCGTTGGA | TTTAATGTCGATATTCTCCTCTTCGA |
| F5H | CGTGGAAGGACCCGGATAGT | TCGCTGCCGCCTATGG |
| HMGS | GGGATGGGCGTTATGGACTT | GCAGGGCCTTCTGCATAAAC |
| HMGR | TGGAGCTCTTGGCGGTTTT | CAATGAACACGGCAGAGACAA |
| MVK | GCTGGTTGCTGGTGTATCAGAA | CACGGCTGCCATTGCA |
| MDD | CACGGGCATGCATGAGAGT | ACAACCTCGGCTCTGTGCTT |
| IPP | GCCTGGTGCGATACAGATTCA | CCGTCGATGCTGCCAAA |
| CAS | CCGGATTTGGTTATCCACAGA | GAGGCTGCGGGAACAATGT |
| KR | CCTCAGGAGGCGATCCAAT | CGGGTGCTCTGCGTTCTT |
| OKS | TCCGCGCCACCAACA | TGCGATCGAACTCTTCTTGAG |
| UGT | CCAGGGACATGGCGAAAC | CCGGGAGGCCAAATGC |
| GAPDH | TTCTGTTGAGGAAGGCATCCA | GCAACCCTGAAGCCCAAAG |

**Table 1: List of Primers for Quantitative RT PCR**
